# Fused Imidazotriazole-Based Therapeutics: A Multidisciplinary Study Against Diabetes-Linked Enzymes Alpha-Amylase and Alpha-Glucosidase Using In Vitro and In Silico Methods

**DOI:** 10.3390/ph18091333

**Published:** 2025-09-05

**Authors:** Manal M. Khowdiary, Shifa Felemban

**Affiliations:** Department of Chemistry, Faculty of Applied Science, University College-Al Leith, University of Umm Al-Qura, Makkah 21955, Saudi Arabia

**Keywords:** diabetes mellitus, imidazo-triazole, molecular docking, dynamic simulation, DFT-ADMET

## Abstract

**Background/Objective:** The present study reports the design, synthesis, and biological evaluation of novel imidazo-triazole derivatives as potential antidiabetic agents. **Methods:** The novel series was synthesized by treating amino-triazole bearing carboxylic acid with substituted 2-bromo acetophenone and was biologically compared with acarbose under in vitro analysis. **Results:** Structure–activity relationship (SAR) analysis revealed that among these compounds, remarkable activity was shown by compound **5** (having three hydroxyl substituents) with IC_50_ value of 6.80 ± 0.10 and 7.10 ± 0.20 µM for α-amylase and α-glucosidase in comparison to reference drug acarbose. To support experimental findings, computational investigations including molecular docking, pharmacophore modeling, molecular dynamics simulations, density functional theory (DFT), and absorption distribution metabolism excretion and toxicity (ADMET) profiling were employed. These studies confirmed the stability of ligand–protein interactions and provided insights into electronic and reactivity features governing enzyme inhibition. **Conclusions:** Collectively, the integration of in vitro and in silico approaches underscores the potential of novel imidazo-triazole scaffolds as promising leads for the development of safer and more effective therapeutics against diabetes mellitus.

## 1. Introduction

Diabetes mellitus (DM) is a persistent disease characterized by elevated blood sugar levels and occurs from an imbalance in the secretion of insulin, insulin activity, or both. The prolonged condition of high blood sugar is linked with the malfunction of organs especially the eyes, kidneys, heart, and capillaries [1,2,3,4]. DM is categorized into two major classes: Type-1 DM (insulin-reliant diabetes mellitus) and Type-2 DM (non-insulin reliant diabetes mellitus). In type-1 DM, the secretion of insulin by pancreatic cells is defected resulting in an elevation of the blood sugar level. This autoimmune disorder develops in a few weeks and is characterized by thirst, urination, blurred vision, and fatigue [5,6,7,8]. More than 90% cases are of type-2 DM, which is caused mainly due to a lower secretion of insulin by pancreatic beta cells (responsible for carbohydrate breakdown) and results in hyperglycemia [5]. Among population affected by DM, 50% are unaware of their disease. The frequency of diabetic patient tolerance is predicted to be 7.5% in 2019 and expected to reach 8.0% by the year 2030 and 8.6% by 2045 [9,10]. The natural history of type-2 DM showed that it most commonly occurs in adults due to an unhealthy lifestyle and also due to insulin resistance or less production. Two primary enzymes, α- amylase along with α-glucosidase are involved in glucose metabolism which leads to hyperglycemia or post prandial-hyperglycemia after a meal. Inhibitors of both enzymes already present are acarbose and miglitol, reducing blood sugar levels with considerable side effects [11,12].

Compounds containing heterocyclic moiety are considered to be promising scaffolds in pharmaceuticals. Among these moieties, triazole is considered to be important in this case, especially when fused with the imidazole ring. Furthermore, imidazole and triazole bearing drugs were found to possess several bioactive properties such as anticancer [13,14], antibacterial [15,16], antifungal [17,18], anti-diabetic [19,20], and antiviral [21,22]. These compounds have shown exceptional inhibition against alpha-amylase along with alpha-glucosidase making them lead candidates for the treatment of DM [23,24,25,26]. In our study we have designed a fused imidazole–triazole compound with highly interactive substituents to evaluate their enhanced anti-diabetic properties (against alpha-amylase and alpha-glucosidase). Fused imidazo–triazole frameworks remain largely underexplored in the context of antidiabetic research, despite a growing body of evidence demonstrating their broad-spectrum bioactivities in other therapeutic areas, including antimicrobial, anticancer, anti-inflammatory, and antiviral applications. The structural fusion of imidazole and triazole rings integrates two pharmacologically privileged scaffolds into a single rigid heterocyclic system, which not only enhances molecular stability but also provides multiple hydrogen-bonding and π–π interaction sites that are critical for enzyme inhibition. By underscoring the unique potential of this fused structure, our study highlights the opportunity to exploit synergistic pharmacophoric features that may lead to improved binding affinity, selectivity toward carbohydrate-hydrolyzing enzymes, and favorable drug-like properties compared to conventional heterocycles.

**Figure 1:**
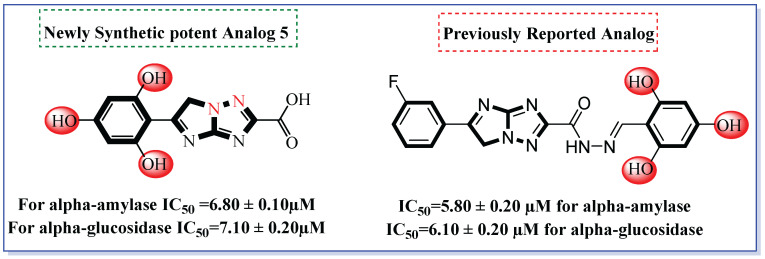
Rationale of current research work.

Figure 1 presents a comparison between the newly synthesized imidazo–triazole derivative (analog 5) and a previously reported scaffold in terms of their inhibitory activities against α-amylase and α-glucosidase. The new compound introduces a fused imidazo–triazole core that broadens the chemical diversity of antidiabetic scaffolds. The structural comparison highlights the distinct substitution pattern of analog 5, particularly the multiple hydroxyl groups, which may contribute to enhanced binding interactions and improved pharmacological potential. This side-by-side illustration underscores the novelty of the fused heterocyclic framework while situating its bioactivity within the context of established analogs. The rationale of the current research study is presented in Figure 1, which represents the previously reported triazole [20] based analog and newly synthesized potent compound.

## 2. Results and Discussion

### 2.1. Synthetic Route

**Scheme 1:**
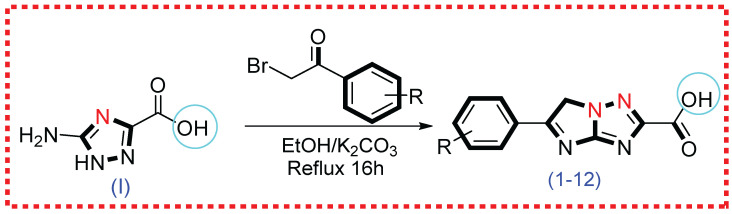
Synthetic procedure adopted for the synthesis of imidazo-triazole based derivatives (**1–12**).

Imidazole–triazole bearing derivatives (**1–12)** were synthesized via a single step by treating two mmol of amino-triazole bearing carboxylic acid with varied one mmol of substituted 2-bromo acetophenone. This reaction was carried out using the solvent ethanol (15 mL). A small amount potassium carbonate was added to catalyze the reaction. The reaction mixture was kept under continuous stirring and reflux for 16 h (Scheme 1). At the initial stage, the synthetic confirmation of all the synthesized compounds was achieved via thin layer chromatography (TLC). A mixture of ethyl acetate and n-hexane in 1:3 was used as the mobile phase for TLC carried out using silica gel plates. After the single spot confirmation of the compounds, solvent was evaporated under low pressure and solid residue was collected. To achieve purification, all the synthesized compounds were washed with n-hexane. Different characterization techniques (^1^H-NMR, ^13^C-NMR, and HERI-MS), were implemented for structural validation of the synthesized derivatives. Synthetic route for fused imidazo-triazole based derivatives (**1–12**) is given in Scheme 1.

Structural confirmation of the synthesized compounds was achieved via spectroscopic analysis by conducting ^1^H-NMR, ^13^C-NMR, and mass spectrometry. ^1^H-NMR spectra were obtained by using the solvent DMSO at operating frequency of 600 MHz. For representative compound **5**, a singlet peak was observed at chemical shift of 12.65 ppm for one proton of carboxylic group. A singlet peak was observed at chemical shift of 10.43 ppm for one proton of the OH group substituted at *para* position of the phenyl ring. A singlet peak was observed at chemical shift of 10.26 ppm for two protons of the OH group at two and six-positions of the phenyl ring. Another singlet peak was observed at chemical shift of 7.16 ppm for two protons of phenyl ring at three and five-position. A singlet peak was observed at chemical shift of 4.57 ppm for two protons of the imidazole ring. The detailed ^1^H-NMR spectrum of compound **5** is presented in Figure S12.

^13^C-NMR spectrum was obtained by using the solvent DMSO at an operating frequency of 150 MHz. The ^13^C-NMR spectrum of compound **5** showed distinct signals corresponding to various carbon environments within the molecule. The signal appeared at chemical shift of 174.9, 163.5, 163.4, 163.3, 162.8, 157.4, 157.2, 111.3, 108.9, 108.5, and 55.2 ppm. These chemical shift assignments correlate well with the proposed structure of compound **5** and confirm the integrity of the synthesized molecule. The detailed ^13^C-NMR spectrum is presented in Figure S13.

HR EIMS was also conducted for representative compound **5**. The *m*/*z* calculated for C_11_H_8_N_4_O_5_ [M]^+^ was 275.21 and was found to be 275.17. HREI-MS spectrum of representative compound **5** is given in Figure S14.

### 2.2. Biological Efficacy

The biological evaluation was carried out to assess inhibitory potential against diabetes-related target enzymes, α-amylase along with α-glucosidase. The inhibitory activity was determined in terms of IC_50_ values across different concentrations, with all experiments performed in triplicate to ensure reproducibility. Notably, all compounds demonstrated significant enzyme inhibition, exhibiting superior activity in comparison with the reference standard drug acarbose with IC_50_ = 9.40 ± 0.20 µM and 9.80 ± 0.30 µM for both enzymes, respectively. The analogs showed various levels of effectiveness from excellent to moderate depending upon the nature and position of substituent attached. Table 1 represents various groups with altering values of suppression.

#### Structure–Activity Relationship (SAR)

The structure–activity relationship (SAR) analysis revealed that hydroxyl-substituted derivatives were the most potent α-amylase and α-glucosidase inhibitors, highlighting the crucial role of hydrogen bonding and electron-donating substituents in enhancing activity. Among them, analog **5** emerged as the most active compound, exhibiting IC_50_ values of 6.80 ± 0.10 µM against α-amylase and 7.10 ± 0.20 µM against α-glucosidase. The strong inhibitory activity is attributed to the presence of three hydroxyl groups positioned at both *ortho* and *para* sites, which engage in extensive hydrogen-bonding interactions with the catalytic residues, while their electron-donating effects further activate the aromatic ring, collectively stabilizing the ligand–enzyme complex. Docking studies confirmed that these hydroxyl substituents participate in polar interactions within the active pocket, reinforcing the in vitro findings.

Similarly, analog **7**, with *ortho-* and *para-*hydroxyl substitution alongside a *meta*-methyl group, also displayed potent inhibition (IC_50_ = 7.10 ± 0.30 µM and 7.70 ± 0.20 µM for α-amylase and α-glucosidase, respectively). The electron-donating effect of hydroxyl groups enhanced ring activation, while the hydrophobic contribution of the methyl group supported additional stabilization within nonpolar regions of the binding pocket [27]. Analog **10**, bearing a *para*-hydroxyl and *meta*-methyl substitution, showed comparable activity (IC_50_ = 8.10 ± 0.20 µM for both enzymes), driven by hydrophilic interactions of the hydroxyl moiety with polar residues and hydrophobic contacts of the methyl group with the nonpolar regions. Analog **9** also demonstrated strong dual inhibition (IC_50_ = 9.20 ± 0.10 µM and 9.70 ± 0.10 µM), substitution of two methyl groups on the *meta* site can increase hydrophobic interaction with non-polar regions of enzyme active site.

Halogen-substituted derivatives showed moderate to good activity. For instance, analog 1, bearing *para-*fluoro and additional methyl substituents, inhibited both enzymes effectively (IC_50_ = 9.30 ± 0.10 µM and 9.90 ± 0.10 µM), where the fluorine atom, due to its small size and high electronegativity, fit favorably into the enzyme pocket and engaged in stabilizing hydrophobic contacts [28]. Among chlorine derivatives, analog **8** (IC_50_ = 11.40 ± 0.20 µM and 12.10 ± 0.50 µM) outperformed analog **11**, reflecting the combined effect of chlorine substitution and *meta-*methyl groups enhancing hydrophobicity and enzyme interactions.

In contrast, bulky halogen substitutions reduced potency [29]. Analog **6** (*para*-bromo, IC_50_ = 20.30 ± 0.40 µM and 21.10 ± 0.40 µM) and analog **4** (*ortho-*bromo, IC_50_ = 21.40 ± 0.10 µM and 22.10 ± 0.10 µM) displayed significantly weaker inhibition, likely due to steric hindrance that disrupted optimal enzyme binding. Nitro-substituted analogs demonstrated variable activity: analog **3** (IC_50_ = 19.10 ± 0.20 µM and 19.70 ± 0.20 µM) was less potent compared to analog **12** (IC_50_ = 17.50 ± 0.60 µM and 18.10 ± 0.20 µM), with the difference explained by the electron-withdrawing nature of nitro groups and ring activation effects contributed by methyl substitutions.

**Figure 2:**
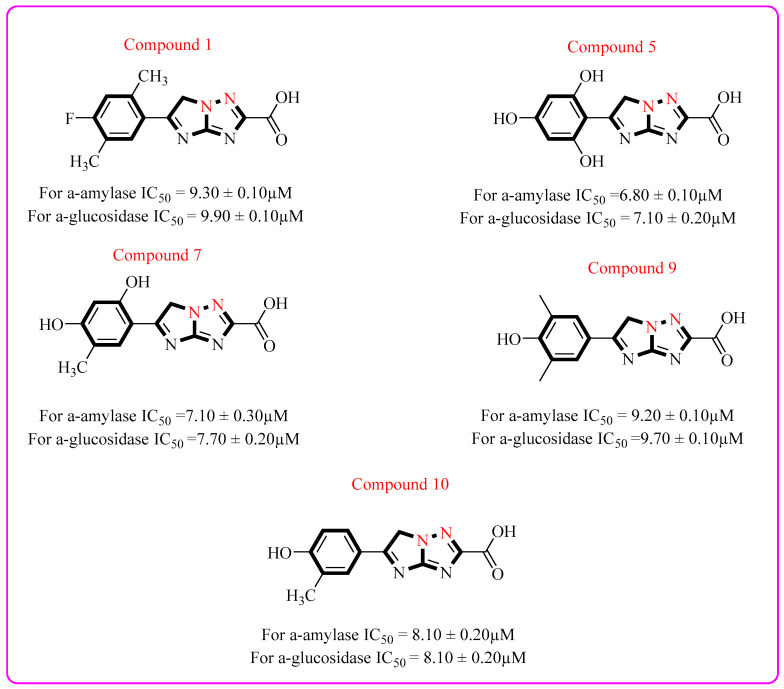
Inhibitory profile of analog **1**, **5**, **7**, **9**, and **10** against alpha-amylase and alpha-glucoside.

Overall, both in vitro enzyme inhibition assays and in silico docking analyses consistently highlighted analogs **5, 7**, and **10** as the most promising anti-diabetic candidates. Their superior activity is driven by the synergistic contribution of hydrogen bonding, electron-donating effects of hydroxyl groups, and stabilizing hydrophobic interactions within the active sites of α-amylase and α-glucosidase. These findings underscore the rational design strategy of combining polar and hydrophobic substituents to achieve balanced interactions, ultimately leading to a potent dual inhibition comparable or superior to the reference drug acarbose. The SAR trends of the most active analogs are summarized in Figure 2.

### 2.3. Molecular Docking

**Figure 3:**
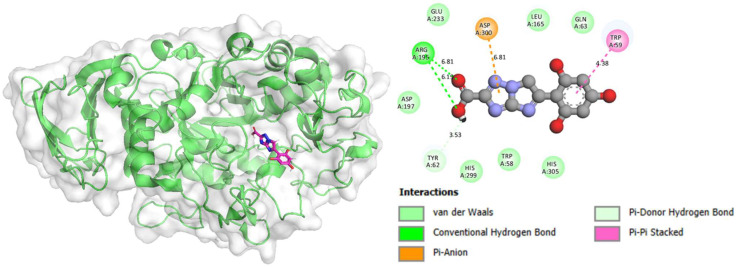
Docking interaction of compound **5** targeting alpha-amylase.

**Figure 4:**
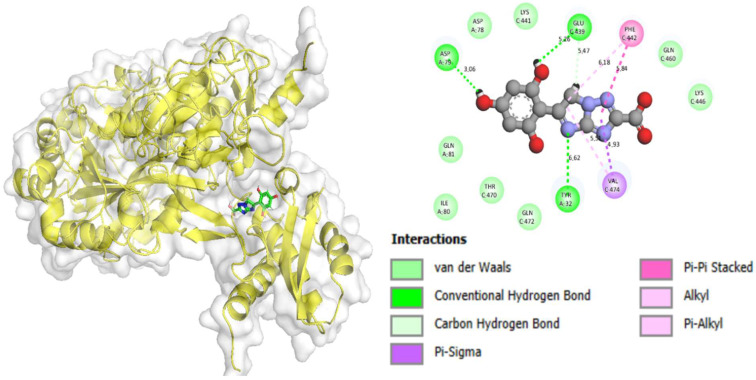
Docking assessment of compound **5** for alpha-glucosidase.

Molecular modeling is a frequently employed computational tool in drug designing. Docking has become integral part of computer aided drug designing and discovery [30,31,32,33,34]. The primary objective of molecular docking is to investigate binding interactions between small molecules (ligands) and large macromolecules (proteins). Docking is basically an in silico technique performed by using software such as AutoDock Vina (1.5.7), DSV (2024), and PyMol (Anaconda3). Molecular docking functions include: evaluating the key interactions between ligands and protein active sites, confirming the structure–activity relationship, and identifying key amino acid residues like hydrogen bonding, Van der Waals interaction, and electrostatic contacts. Our research group has executed molecular modeling studies on a range of moieties in relation to both targeted enzymes. The in silico results of most potent analogs **5**, **7**, and **10** are illustrated in Figure 3 and Figure 4 and Supplementary Figures S1–S4.

The molecular docking studies were carried out to investigate the binding interactions of the synthesized compounds with the target enzymes α-amylase and α-glucosidase. The crystal structures of human pancreatic α-amylase (PDB ID: 1B2Y, resolution: 3.20 Å) and α-glucosidase (PDB ID: 3W37, resolution: 1.70 Å) were retrieved from the Protein Data Bank (PDB). Prior to docking, water molecules and heteroatoms were removed, while essential cofactors and catalytic residues were retained. The protein structures were energy minimized and prepared using AutoDock Vina, with hydrogen atoms added and protonation states adjusted at physiological pH (7.4). The docking grid was defined to encompass the enzyme’s active site, using coordinates derived from the co-crystallized ligand in the reference structure. The grid center and dimensions (x × y × z Å) were selected to fully include all known catalytic residues and accommodate ligand flexibility. A grid box was constructed to define the active binding site of the enzyme, ensuring that the ligand explores the relevant binding pocket during the docking process. The grid box dimensions were set as follows:

- Center coordinates: (x = 3.625794, y = 64.910854, z = 68.89273);
- Grid box size: (x = 20, y = 20, z = 20).

The ligands were drawn in ChemDraw (15.1), converted to 3D structures, and energy minimized. Validation of the docking protocol was achieved by re-docking the co-crystallized ligand into the binding pocket and calculating the root mean square deviation (RMSD) between the docked and experimental poses. An RMSD value ≤ 2.0 Å was considered acceptable, confirming the reliability of the docking procedure. Docking poses were analyzed based on binding energy values and key interactions, including hydrogen bonding, hydrophobic contacts, and π–π stacking, using visualization tools PyMOL and Discovery Studio Visualizer (2024). The docking results were correlated with experimental IC_50_ values to strengthen the structure–activity relationship (SAR) interpretation.

The substituents in compound **5** significantly affect its binding affinity and orientation by forming specific non-covalent interactions within the binding pocket. For example, hydroxyl (–OH) groups act as hydrogen bond donors/acceptors, stabilizing the ligand through interactions with key residues such as Glu240 and Asp197. Aromatic or heteroaromatic substituents enhance binding via π–π stacking or π–cation interactions with residues like Tyr62 or His201, improving affinity. Hydrophobic substituents (e.g., alkyl or phenyl groups) occupy nonpolar pockets, strengthening Van der Waals contacts with residues like Val98, Leu162, and Ile235. The electron-withdrawing or electron-donating nature of substituents further modulates hydrogen-bond strength and electronic distribution of the ligand, influencing conformational fit and interaction stability. Overall, substituents guide both the orientation of the ligand and the strength of intermolecular interactions, thereby determining the binding efficiency and biological activity of the compound.

Results of the in silico study for compounds **5**, **7,** and **10** show binding poses in enzymes active sites. Both 2D and 3D images demonstrate fittings of compounds into pockets of both amylase and glucosidase, forming bonds with amino acid residues, especially for Glu 248. Analog **5** was found to bind with the target enzymes alpha-amylase and alpha-glucosidase by engaging with the amino acids using the hydroxyl substitutions. The hydroxyl group is reported as a strong interactive moiety due to the virtue of hydrogen bond formation and other interactions [35]. The docking images for compound **5** show its interactions with the active site of both enzymes highlighting interactions like hydrogen bonds represented by green dash lines while other interactions, like hydrophobic and π–π interactions are shown by pink and purple colors. In addition, aromatic rings may align with aromatic residues confirming aromatic interaction π–π stacked, arene–arene, arene–H, and arene–cation. Docking images illustrate that compound **5** is more potent than **7** and **10** due to the strong interactions of ligand with the enzymes’ active site**,** where the 3D structure explains how well ligand fits in the active site of both enzymes represented by the net structure, while different interactions in the 2D diagram explain how it shows strong bonding with the protein active site like it does in the lock and key model. Supplementary Figures S1 and S2 explain the docking profiles of compound **7** for alpha-amylase and alpha-glucosidase, showing how it fits into the pocket of both enzymes by using 3D and 2D images. The result shows that compound **7** is well fitted into the pockets of both enzymes shown by different interactions of hydrophobic and hydrophilic nature. Likewise, Supplementary Figures S3 and S4 demonstrate the activity of compound **10** for both enzymes and shows its interactions with the enzymes’ active sites. Molecular docking studies confirm that compound **5** is the most potent when compared to **7** and **10** and suggests its higher potency and better fit with enzymes’ active sites. Detailed docking outcomes, including type of receptor, interactions, binding distance, and docking score of potent analogs against targeted enzymes are presented in Table 2.

### 2.4. Pharmacophore Mapping

**Figure 5:**
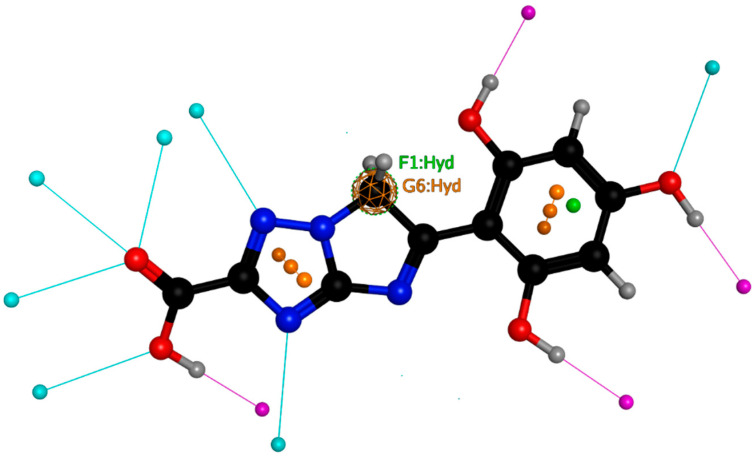
Pharmacophore model of potent compound **5**.

The term pharmacophore was first proposed by Ehrlich in 1909, who described the pharmacophore as ‘a molecular framework that carries (phoros) the essential features responsible for a drug’s (pharmacon) pharmacological activity. Pharmacophore modeling is one of the basic computational techniques used for drug designing. The primary objective of pharmacophore modeling is to examine the interaction between protein and ligand and also to confirm the results of molecular docking and simulation. It helps researchers to understand molecular interactions, to identify potential drug candidates, and to optimize molecules. Its applications consist of virtual screening, ADME-TOX, side effect modeling, and target identification. Key features of pharmacophore modeling involve H-bond donors, H-bond acceptors, aromatic interactions, hydrophobic regions, and ionic features. The process of pharmacophore modeling consists of following steps: data collection, molecular alignment, feature extraction, model generation, validation, and virtual screening (Figure 5).

Pharmacophore model of compound **5** illustrates the key structural features of its higher potency against both targeted enzymes alpha-amylase and alpha-glucosidase. The colored spheres and sticks indicate different atoms: carbon (black), nitrogen (blue), oxygen (red), hydrogen (white/gray), halogens (cyan/magenta), and sulfur (yellow/orange). The highlighted regions labeled F1:Hyd and G6:Hyd denote specific hydrogen-bond interactions between the ligand and amino acid residues of the receptor, critical for stabilization within the binding pocket. These interactions, along with the aromatic stacking and electron-rich heteroatoms, suggest that the compound achieves strong binding affinity and specificity, making it a promising candidate for biological evaluation as an inhibitor or modulator of the target protein.

### 2.5. Molecular Dynamic (MD) Simulation Studies

**Chart 1:**
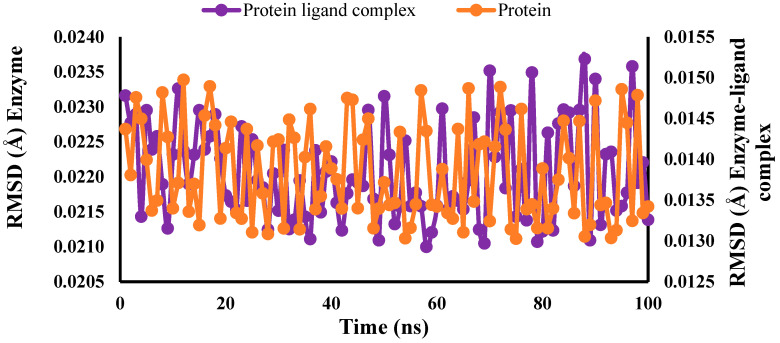
MD simulation of compound **5**. The *x*-axis represents the time (nanosecond, ns) and the *y*-axis shows root mean square deviation, RMSD (Å) for enzyme (**left**) and for enzyme–ligand complex (**right**).

In silico simulation is adopted to explore the behavior of ligand–protein interaction and to confirm the results of molecular docking. Unlike molecular docking, which explains binding poses, MD simulations allow for dynamic behavior of protein–ligand complexes. Molecular dynamic simulation was carried out by using different software such as AMBER, CHARMM, LAMMPS, and NAMD. MD simulation involves the following steps: structure preparation, solvation and ionization, energy ionization, energy RUN and ionization, and energy dynamics. Molecular dynamic simulation is a useful computational technique; however, it does have some limitations like its expensive cost and its handling requires specific techniques like Meta dynamics and accelerated MD (Chart 1). A molecular dynamics simulation of compound **5** was conducted in a defined period of time to assess its stability and interaction. The RMSD graph shows that the complex attains equilibrium quickly and retains stability throughout the simulation, indicating stable interactions. A consistently high number of hydrogen bonds indicates stable and excellent interactions, thus supporting docking predictions; however, the protein–ligand complex (purple) shows less fluctuation and more stability. Furthermore, protein (orange) shows that it is quite stable but more flexible than the ligand complex (purple) and seems to stabilize the enzyme structure.

Chart 1 shows an RMSD (root mean square deviation) plot comparing enzyme and enzyme–ligand complex under molecular dynamics (MD) simulation of a protein–ligand complex. RMSD measures structural deviations over time, reflecting the stability and flexibility of the system. From the plot, both systems fluctuate within a range of approximately 0.5 to 2.5 Å, suggesting overall structural stability. The protein trajectory shows slightly higher fluctuations compared to the protein–ligand complex, indicating that the ligand or substituent in that system induces more conformational flexibility within the protein binding site. Conversely, the protein–ligand complex demonstrates relatively lower RMSD values, implying a more stable binding orientation and stronger intermolecular interactions. This means the nature of the substituent in the ligand directly influences complex stability: polar or hydrogen-bonding substituents can anchor the ligand firmly (lower RMSD), whereas bulky or less interactive substituents may destabilize the binding conformation (higher RMSD).

### 2.6. Density Functional Theory (DFT)

Density functional theory is an in silico technique used to calculate different electronic properties of the compounds by using Gaussian 09 quantum chemical software [36,37,38]. It provides insight into reactivity, structure, and molecular properties. The DFT model helps to confirm the hydrophobic and hydrophilic interactions of the compounds like hydrogen-bonding, π–π stacking, and Van der Waals interactions. The results of molecular docking and MD simulations were supported by performing DFT analysis as it precisely calculates HOMO-LUMO energies and electrostatic potential maps. In addition, interaction between analog and enzyme was estimated by evaluating electrostatic potential distribution. The geometrics of compounds were analyzed by using Gauss View 5.0 [39,40].

In the present study, the density functional theory (DFT) calculations for geometry optimization and electronic property analysis of the most active compounds were carried out using the B3LYP functional with the 6-31G(d,p) basis set. This level of theory was selected because it has been extensively reported in the literature as a reliable and cost-effective method for predicting the optimized geometries, frontier molecular orbitals, and reactivity descriptors of heterocyclic derivatives. Previous studies have demonstrated that B3LYP/6-31G(d,p) provides accurate results in terms of electronic distribution, stability, and reactivity trends, while maintaining computational efficiency. Therefore, employing this level of theory ensures both consistency with the prior literature and reliability in interpreting the chemical reactivity and interaction potential of the synthesized analogs.

#### 2.6.1. Molecular Electrostatic Potential Map

**Figure 6:**
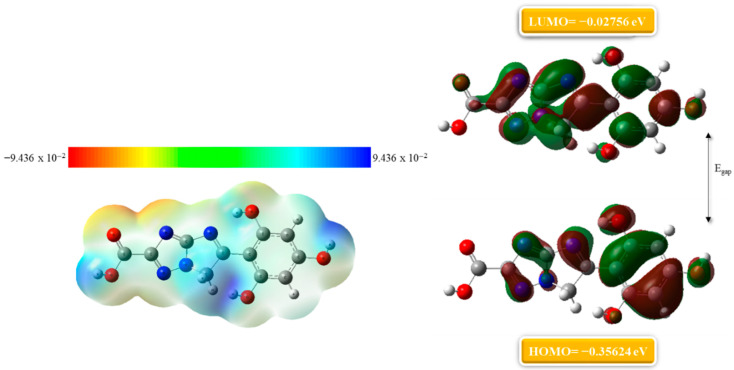
Molecular electrostatic potential and FMO analysis of compound **5**.

Molecular electrostatic potential plays a vital role in the calculation of charge distribution within molecules, which directly explains its interaction with target sites such as enzymes, receptors, and nucleic acids. In addition to that, molecular electrostatic potential (MESP) also helps to identify the nucleophilic and electrophilic sites of molecules, which correspond to binding sites [41,42]. MESP maps highlight electron rich sites (electrophilic attack center) with red regions and electron deficient site (nucleophilic attack center) with blue regions. The MESP of compound **5** is illustrated in the left corner of Figure 6 where an intense red region shows highly negative electrostatic potential (hydrogen-bond acceptor sites) due to the presence of three hydroxyl groups, while blue regions suggest hydrogen-bond donor sites due to the presence of N–H bonds. Hydrophobic interactions are shown by green zones highlighting π–π interactions. Compared to compound **5**, the MESP of compound **7** shows slightly less charge distribution, meaning it may have fewer electrostatic interactions while it can still participate in hydrophobic and hydrophilic interactions with red regions having negative electrostatic potential and blue regions having positive electrostatic potential, as illustrated in Supplementary Figure S5 in the left corner.

#### 2.6.2. Frontier Molecular Orbitals (FMO) Analysis

Frontier molecular orbitals analysis was performed to examine the profile of a molecule by assessing its highest occupied energy level (HOMO) and lowest unoccupied energy level (LUMO) upon exposure with a protein active site [43]. The energy gap between the HOMO and LUMO predicts molecular stability and reactivity. Frontier orbital analysis of compound **5** is examined using densities of HOMO–LUMO. Findings show that the HOMO is localized at the right corner of the compound with energy of −0.35624 and the LUMO is delocalized on the entire molecule with energy of −0.02756 eV. The stability of the compound is best explained by the energy gap between the HOMO and LUMO, a low energy gap of (−0.32868 eV) means it is more stable and it can perfectly fit into the enzyme active site, as shown in Figure 6.

Likewise, FMO analysis of compound **7** is depicted in Supplementary Figure S5 which explains the interactions of ligand with the protein active site by looking into energy gaps between molecular orbitals. The calculated energy values for the HOMO and LUMO are −0.33716 eV and −0.03548 eV. The results of FMO analysis show that the energy gap (−0.37264 eV) between the HOMO and LUMO of compound **7** is greater than gap of compound **5** which predict that compound **5** is the most potent as it perfectly fits into the enzyme active site.

This Figure 6 shows the frontier molecular orbital (FMO) analysis and molecular electrostatic potential (MEP) mapping of compound **5**, highlighting its electronic properties and reactive behavior. The left side displays the MEP surface, where different colors represent regions of varying electron density: red and yellow regions indicate electron-rich areas prone to electrophilic attack, while blue regions denote electron-deficient zones favorable for nucleophilic interactions. On the right side, the Highest Occupied Molecular Orbital (HOMO, −0.35624 eV) and Lowest Unoccupied Molecular Orbital (LUMO, −0.02756 eV) distributions are shown. The HOMO is primarily localized over heteroatoms and aromatic π-systems, suggesting potential sites for electron donation, whereas the LUMO is spread over electron-deficient regions, identifying sites for electron acceptance. The relatively small HOMO–LUMO energy gap implies high chemical reactivity and favorable charge transfer potential, making the compound a strong candidate for biological interactions, particularly in binding to active sites of enzymes or receptors.

### 2.7. Absorption Distribution Metabolism Excretion and Toxicity (ADMET) Analysis

**Chart 2:**
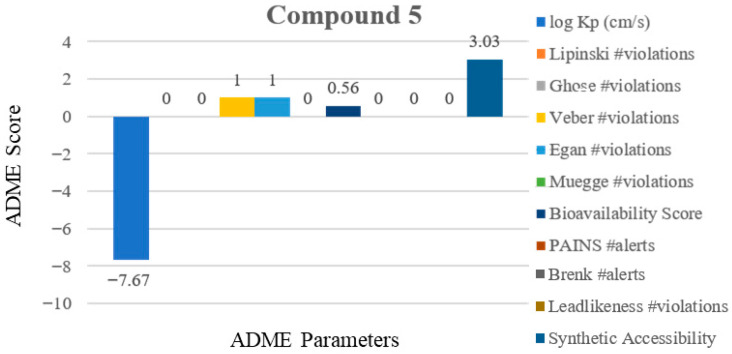
ADMET profile of compound **5**. *x*-axis shows different ADME parameters and *y*-axis shows ADME score of different parameters.

**Figure 7:**
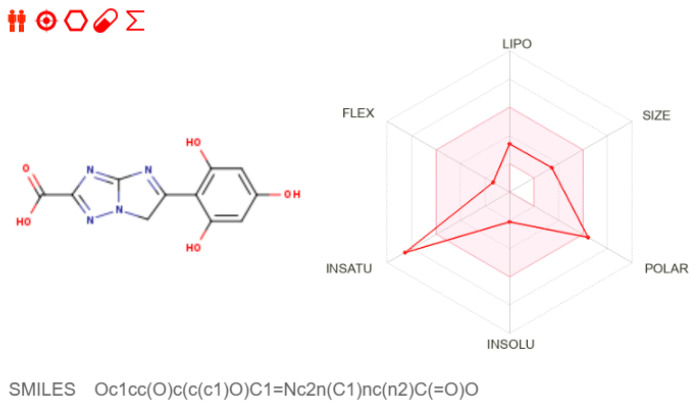
SMILES notation for analog **5**.

**Figure 8:**
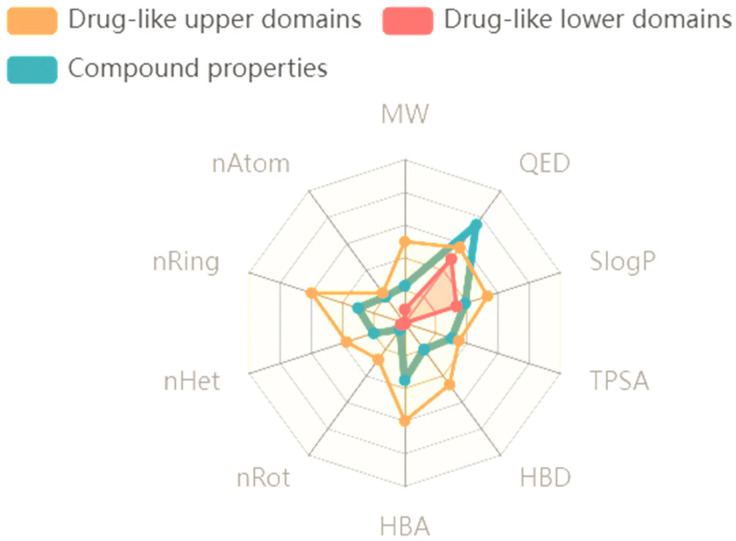
Compound **5** domain in comparison to drug like upper and lower domain characteristics.

ADMET analysis is a frequently used computational tool. The acronym stands for absorption, distribution, metabolism, excretion, and toxicity and is a computational tool used for evaluating lead drug candidates. It predicts how well a compound behaves in the body, aiming to diagnose issues before further proceeding to clinical trial. Absorption predicts how well a compound is absorbed into the blood crossing the blood body barrier (BBB), distribution looks at how it is distributed into body, metabolism describes how it is metabolized into body, especially in the liver. Excretion looks up how the drug and waste products are excreted through body via urinary system. Toxicity evaluates whether it is safe to use as a drug or not. ADMET analysis is basically carried out by using software like Swiss ADME (https://www.swissadme.ch/). One of the fundamental tools in ADMET analysis is the Lipinski rule of 5 which explains drug likeness by the following parameters: molecular weight, hydrogen-bond donor, and acceptors. Furthermore, skin permeability is explained by the log kp rule with +3> shows that a drug is fat soluble while −3< shows that it is water soluble. Chart 2 and Supplementary Charts S1 and S2 shows that there is no violation of ADME rules by active analogs 5, 7, and 10. Results show that compound **5** is the most potent as it follows all rules of ADME. The SMILES notation and boiled egg representation for analog 5 are depicted in Figure 7 and Supplementary Figure S6. The molecular weight and dose value distribution for analog 5 are represented in Supplementary Charts S3 and S4. The drug like domain of the potent analogs is shown in Figure 8 and Supplementary Figures S7 and S8. Detailed ADMET properties are illustrated in Table 3 and Supplementary Tables S1 and S2.

The drug-likeness and ADME profiling of compound **5** are summarized in Chart 2. The compound shows a skin permeability (log Kp) value of –7.67 cm/s, which indicates low transdermal diffusion and is consistent with orally active drug candidates. Importantly, compound **5** did not violate any of the major drug-likeness filters, including Lipinski, Ghose, Veber, Egan, and Muegge rules, suggesting favorable physicochemical properties for oral bioavailability. The bioavailability score was predicted to be 0.56, which is considered moderate and supportive of acceptable absorption potential. Additionally, no PAINS (Pan-Assay Interference Compounds) alerts were detected, indicating a low risk of assay interference and suggesting the compound is structurally suitable for further biological evaluation. Overall, the ADME and drug-likeness assessment highlights compound **5** as a promising candidate with favorable pharmacokinetic and safety-related properties.

## 3. Materials and Methods

### 3.1. Materials

To synthesize imidazole–triazole hybrid-based derivatives, various chemicals and reagents were acquired from Sigma Aldrich, St. Loius, MO, USA. Kieselgel 60,254, E TLC plates for monitoring the reaction were sourced from Merck, Hamburg, Germany. TLC plates were examined under a UV lamp of wavelength 254 and 365 nm. For novel compounds, structural validation spectroscopic technique NMR was performed using a NMR Bruker AM instrument (600 MHz). Peaks splitting pattern was analyzed and observed as singlet, dt, doublet of doublet, triplet and multiplicity to calculate the coupling constant values (J) in hertz (Hz). (HREI-MS) high resolution electron mass spectra of these compounds were obtained using a Finnigan MAT-311A mass spectrometer (Munich, Germany).

### 3.2. Methodology for the Synthesis of Imidazo–Triazole Conjugates (***1**–**12***)

To synthesize novel imidazo-triazole based compounds (**1**–**12**), a single route was adopted. Amino-triazole bearing carboxylic acid (2 mmol) was treated with 2-bromo acetophenone (1 mmol) in ethanol (15 mL) as a solvent. A catalytic amount of potassium carbonate was also added and the mixture was refluxed for 16 h. This results in the successful synthesis of novel derivatives which were confirmed via TLC. Once the reaction was completed, solvent was removed under low pressure then the synthesized product in a solid state was obtained which was rinsed with *n*-hexane and subsequently dried to obtain purified products. For structural validation spectroscopic techniques NMR and HEIR-MS were carried out.

### 3.3. Spectroscopic Analysis

5-(4-Fluoro-2,5-dimethylphenyl)-6*H*-imidazo{1,2-*b*]{1,2,4]triazole-2-carboxylic acid (**1**)

Yield: 84%; m.p: 215–223 °C. ^1^H-NMR (600 MHz, DMSO-*d*_6_): *δ* 12.72 (s, 1H, H-OH), 7.64 (s, 1H, Ar-H), 6.81 (s, 1H, Ar-H), 4.55 (s, 2H, H-imidazole), 2.45 (s, 3H, H-aliphatic), 2.31 (s, 3H, H-aliphatic); ^13^C-NMR (150 MHz, DMSO-*d*_6_): *δ* 172.1, 164.2, 163.5, 154.7, 153.1, 137.3, 130.7, 125.1, 121.2, 116.1, 56.2, 19.1, 14.2; HREI MS: *m*/*z* calcd for C_13_H_11_FN_4_O_2_ {M]^+^ 274.26 Found 274.22.

5-(m-Tolyl)-6*H*-imidazo{1,2-*b*]{1,2,4]triazole-2-carboxylic acid (**2**)

Yield: 86%; m.p: 224–227 °C. ^1^H-NMR (600 MHz, DMSO-*d*_6_): *δ* 12.71 (s, 1H, H-OH), 7.82 (dd, *J* = 7.34, 2.24 Hz, 1H, Ar-H), 7.73 (s, 1H, Ar-H), 7.42 (dd, *J* = 7.26 Hz, 1H, Ar-H), 7.25 (dd, *J* = 7.30, 2.26 Hz, 1H, Ar-H), 4.54 (s, 2H, H-imidazole), 2.40 (s, 3H, H-aliphatic); ^13^C-NMR (150 MHz, DMSO-*d*_6_): *δ* 172.2, 164.1, 154.6, 153.1, 138.2, 133.6, 131.1, 129.2, 128.5, 125.1, 56.1, 21.2; HREI MS: *m*/*z* calcd for C_12_H_10_N_4_O_2_ {M]^+^ 242.24 Found 242.20.

5-(3,5-Dimethyl-4-nitrophenyl)-6*H*-imidazo{1,2-*b*]{1,2,4]triazole-2-carboxylic acid (**3**)

Yield: 79%; m.p: 210–213 °C. ^1^H-NMR (600 MHz, DMSO-*d*_6_): *δ* 12.73 (s, 1H, H-OH), 7.84 (s, 2H, Ar-H), 4.47 (s, 2H, H-imidazole), 2.24 (s, 6H, H-aliphatic); ^13^C-NMR (150 MHz, DMSO-*d*_6_): *δ* 174.9, 163.5, 153.4, 151.7, 150.5, 142.8, 133.4, 129.9, 128.5, 125.2, 54.3, 19.7, 19.3 HREI MS: *m*/*z* calcd for C_13_H_11_N_5_O_4_ {M]^+^ 301.26 Found 301.22.

5-(2-Bromophenyl)-6*H*-imidazo{1,2-*b*]{1,2,4]triazole-2-carboxylic acid (**4**)

Yield: 81%; m.p: 234–236 °C. ^1^H-NMR (600 MHz, DMSO-*d*_6_): *δ* 12.72 (s, 1H, H-OH), 7.68 (dd, *J* = 7.23, 2.18 Hz, 1H, Ar-H), 7.55 (dd, *J* = 7.27, 2.24 Hz, 1H, Ar-H), 7.42 (m, 1H, Ar-H), 7.31 (m, 1H, Ar-H), 4.54 (s, 2H, H-imidazole); ^13^C-NMR (150 MHz, DMSO-*d*_6_): *δ* 172.1, 164.2, 154.7, 153.1, 135.2, 134.5, 132.6, 130.1, 127.5, 122.1, 56.2; HREI MS: *m*/*z* calcd for C_11_H_7_BrN_4_O_2_ {M]^+^ 307.11 Found 307.07.

5-(2,4,6-Trihydroxyphenyl)-6*H*-imidazo{1,2-*b*]{1,2,4]triazole-2-carboxylic acid (**5**)

^1^H-NMR (600 MHz, DMSO-*d*_6_): *δ* 12.65 (s, 1H, H-OH), 10.43 (s, 1H, H-OH), 10.26 Yield: 88%; m.p: 239–241 °C. (s, 2H, H-OH), 7.16 (s, 2H, Ar-H), 4.45 (s, 2H, H-imidazole); ^13^C-NMR (150 MHz, DMSO-*d*_6_): *δ* 174.9, 163.5, 163.4, 163.3, 162.8, 157.4, 157.2, 111.3, 108.9, 108.5, 55.2; HREI MS: *m*/*z* calcd for C_11_H_8_N_4_O_5_ {M]^+^ 275.21 Found 275.17.

5-(4-Bromo-3-methylphenyl)-6*H*-imidazo{1,2-*b*]{1,2,4]triazole-2-carboxylic acid (**6**)

Yield: 75%; m.p: 218–220 °C. ^1^H-NMR (600 MHz, DMSO-*d*_6_): *δ* 12.72 (s, 1H, H-OH), 7.61 (s, 1H, Ar-H), 7.58 (d, *J* = 7.24 Hz, 1H, Ar-H), 7.51 (d, *J* = 7.34 Hz, 1H, Ar-H), 4.53 (s, 2H, H-imidazole), 2.33 (s, 3H, H-aliphatic); ^13^C-NMR (150 MHz, DMSO-*d*_6_): *δ* 172.2, 164.2, 154.5, 153.1, 138.1, 132.7, 131.4, 131.3, 127.2, 125.3, 56.1, 23.5; HREI MS: *m*/*z* calcd for C_12_H_9_BrN_4_O_2_ {M]^+^ 321.13 Found 321.09.

5-(2,4-Dihydroxy-5-methylphenyl)-6*H*-imidazo{1,2-*b*]{1,2,4]triazole-2-carboxylic acid (**7**)

Yield: 77%; m.p: 217–219 °C. ^1^H-NMR (600 MHz, DMSO-*d*_6_): *δ* 12.28 (s, 1H, H-OH), 10.23 (s, 1H, H-OH), 9.62 (s, 1H, H-OH), 7.38 (s, 1H, Ar-H), 7.00 (s, 1H, Ar-H), 4.63 (s, 2H, H-imidazole), 2.26 (s, 3H, H-aliphatic); ^13^C-NMR (150 MHz, DMSO-*d*_6_): *δ* 173.5, 163.4, 159.9, 159.4, 153.5, 150.5, 133.4, 118.5, 111.3, 105.5, 55.2, 19.7; HREI MS: *m*/*z* calcd for C_12_H_10_N_4_O_4_ {M]^+^ 273.24 Found 273.20.

5-(4-Chloro-3,5-dimethylphenyl)-6*H*-imidazo{1,2-*b*]{1,2,4]triazole-2-carboxylic acid (**8**)

Yield: 68%; m.p: 215–218 °C. ^1^H-NMR (600MHz, DMSO-*d*_6_): *δ* 12.58 (s, 1H, H-OH), 7.59 (s, 2H, Ar-H), 4.54 (s, 2H, H-imidazole), 3.25 (s, 6H, H-aliphatic); ^13^C-NMR (150 MHz, DMSO-*d*_6_): *δ* 173.5, 162.4, 153.5, 150.5, 136.2, 135.6, 133.4, 129.6, 126.4, 125.2, 57.5, 19.7, 19.3; HREI MS: *m*/*z* calcd for C_13_H_11_ClN_4_O_2_ {M]^+^ 290.71 Found 290.67.

5-(4-Hydroxy-3,5-dimethylphenyl)-6*H*-imidazo{1,2-*b*]{1,2,4]triazole-2-carboxylic acid (**9**)

Yield: 73%; m.p: 234–236 °C. ^1^H-NMR (600 MHz, DMSO-*d*_6_): *δ* 12.71 (s, 1H, H-OH), 8.04 (s, 1H, H-OH), 7.42 (s, 2H, Ar-H), 4.53 (s, 2H, H-imidazole), 2.13 (s, 6H, H-aliphatic); ^13^C-NMR (150 MHz, DMSO-*d*_6_): *δ* 172.1, 164.2, 154.7, 154.5, 153.1, 127.5, 127.3, 126.2, 122.4, 122.3, 56.2, 15.2, 15.1; HREI MS: *m*/*z* calcd for C_13_H_12_N_4_O_3_ {M]^+^ 272.26 Found 272.22.

5-(4-Hydroxy-3-methylphenyl)-6*H*-imidazo{1,2-*b*]{1,2,4]triazole-2-carboxylic acid (**10**)

Yield: 84%; m.p: 224–226 °C. ^1^H-NMR (600 MHz, DMSO-*d*_6_): *δ* 12.72 (s, 1H, H-OH), 9.65 (s, 1H, H-OH), 7.62 (d, *J* = 7.28 Hz, 1H, Ar-H), 7.55 (s, 1H, Ar-H), 7.02 (d, *J* = 7.36 Hz, 1H, Ar-H), 4.52 (s, 2H, H-imidazole), 2.12 (s, 3H, H-aliphatic); ^13^C-NMR (150 MHz, DMSO-*d*_6_): *δ* 172.2, 164.1, 156.5, 154.5, 153.1, 130.4, 126.3, 126.1, 124.5, 115.6, 56.2, 18.2; HREI MS: *m*/*z* calcd for C_12_H_10_N_4_O_3_ {M]^+^ 258.24 Found 258.20.

5-(4-Chlorophenyl)-6*H*-imidazo{1,2-*b*]{1,2,4]triazole-2-carboxylic acid (**11**)

Yield: 70%; m.p: 209–211 °C. ^1^H-NMR (600 MHz, DMSO-*d*_6_): *δ* 12.73 (s, 1H, H-OH), 7.96 (d, *J* = 7.30 Hz, 2H, Ar-H), 7.60 (d, *J* = 7.32 Hz, 2H, Ar-H), 4.53 (s, 2H, H-imidazole); ^13^C-NMR (150 MHz, DMSO-*d*_6_): *δ* 172.1, 164.2, 154.7, 153.1, 136.4, 132.1, 128.7, 128.5, 128.2, 128.1, 56.2; HREI MS: *m*/*z* calcd for C_11_H_7_ClN_4_O_2_ {M]^+^ 262.65 Found 262.61.

5-(4-Nitrophenyl)-6*H*-imidazo{1,2-*b*]{1,2,4]triazole-2-carboxylic acid (**12**)

Yield: 76%; m.p: 225–227 °C. ^1^H-NMR (600 MHz, DMSO-*d*_6_): *δ* 12.72 (s, 1H, H-OH), 8.32 (d, *J* = 7.34 Hz, 2H, Ar-H), 8.04 (d, *J* = 7.28 Hz, 2H, Ar-H), 4.54 (s, 2H, H-imidazole); ^13^C-NMR (150 MHz, DMSO-*d*_6_): *δ* 172.2, 164.3, 154.7, 153.1, 150.1, 140.1, 127.5, 127.4, 127.2, 127.1, 56.2; HREI MS: *m*/*z* calcd for C_11_H_7_N_5_O_4_ {M]^+^ 273.21 Found 273.17.

### 3.4. Assay Protocols of Inhibitory Activity

#### 3.4.1. Alpha-Amylase Inhibition Assay

The in vitro alpha-amylase study was conducted and the enzyme was sourced from Merck (Sigma-Aldrich, Hamburg, Germany). The inhibitory activity of the synthesized compounds against α-amylase was evaluated using a standard protocol with slight modifications [44]. In brief, 250 µL of the test compound (prepared at varying concentrations, 50–250 µg/mL) was mixed with 250 µL of starch solution [1% (*w*/*v*)] and 250 µL of α-amylase solution (1 U/mL). The reaction mixture was incubated at 20 °C for 3 min, after which enzymatic activity was terminated by the addition of 500 µL of dinitrosalicylic acid (DNS) color reagent. The mixture was then heated in a boiling water bath at 85 °C for 15 min, cooled to room temperature for 5 min, and diluted with 4.5 mL of distilled water to a final volume of 6.0 mL. Absorbance was measured at 540 nm using a UV-visible spectrophotometer. A control containing all reagents except the test compound was prepared in parallel, while acarbose was used as the positive control. The percentage inhibition was calculated and all experiments were conducted in triplicate.

#### 3.4.2. Alpha-Glucosidase Inhibition Assay

The in vitro alpha-glucosidase study was conducted and the enzyme was sourced from Merck (Sigma-Aldrich). The α-glucosidase inhibitory activity was determined following a standard microplate-based method [45]. In each well of a 96-well plate, 35 µL of phosphate buffer (pH 6.8), 31 µL of the test compound solution (50–250 µg/mL), and 18 µL of p-nitrophenyl-α-D-glucopyranoside (pNPG, substrate) were added and pre-incubated at 37 °C for 5 min. The reaction was initiated by the addition of 16 µL of α-glucosidase (0.15 U/mL in phosphate buffer), resulting in a total assay volume of 100 µL. After incubation, the reaction was terminated by adding 100 µL of sodium carbonate (200 mM), and absorbance was recorded at 405 nm using a microplate reader. A control without test compound was used as blank, and acarbose served as the standard reference inhibitor. The percentage inhibition was calculated according to the following equation:

$$\%\;inhibition=\frac{\left(Abs\;control\;-\;Abs\;sample\right)}{Abs\;control}\times 100$$

All assays were performed in triplicate to ensure reproducibility.

### 3.5. Molecular Docking Assay Protocols

Protein data bank (PDB) was used as a medium for the retrieval of crystalline structure, optimizing the structure by the removal of water molecules, co-factors, and hetero-atoms and computing hydrogen bonds, charges, and the missing atoms. Synthesized derivatives used for docking studies were prepared and then optimized by the use of a built and ligand preparation module implemented in Discovery Studio 2022 (Dassault Systemes BIOVIA, San Diego, CA, USA)). A gold docking tool was used for docking analysis, ligand preparation involves generating varied tautomer’s, bond order assigning and stereochemistry. Furthermore, the amylase active site was surrounded by the receptor grid choosing centroid of complex ligand (Montbretin A). A radius of 12 Å around the Montbretin A binding site was defined as an enzyme active site. The accomplishment of docking calculations was achieved using the Chem PLP scoring function [46].

### 3.6. Dft Assay

The geometric parameters and energies were computed by density functional theory at the B3LYP/CEP-631G level of theory, using the GAUSSIAN 09W package of the program [47], on geometries that were optimized at a CEP-631G basis set. The high basis set was chosen to detect the energies at a highly accurate level. The atomic charges were computed using the natural atomic orbital populations. B3LYP is the key word for the hybrid functional [48], which is a linear combination of the gradient functionals proposed by Becke [49] and Lee, Yang, and Parr [50], together with the Hartree–Fock local exchange function [51].

## 4. Conclusions

The present study reports on the design, synthesis, and biological evaluation of a series of imidazo-triazole derivatives as potential anti-diabetic agents. Structural confirmation of the synthesized analogs was achieved through spectroscopic techniques, including ^13^C-NMR, ^1^H-NMR, and HR-MS. Among the tested compounds, analog **5** demonstrated the most potent inhibitory activity against both α-amylase and α-glucosidase, with IC_50_ values of 6.80 ± 0.10 µM and 7.10 ± 0.20 µM, respectively. The enhanced activity of analog **5** can be attributed to the presence of three hydroxyl substituents, which enable favorable accommodation within the enzyme active site by establishing multiple hydrogen bond interactions. For comparison, the reference drug acarbose exhibited a relatively weaker inhibition with IC_50_ values of 9.40 ± 0.20 µM and 9.80 ± 0.30 µM against α-amylase and α-glucosidase, respectively. In addition, analogs **7** and **10** also showed promising inhibitory potential, with IC_50_ values of 7.10 ± 0.30 µM and 8.10 ± 0.20 µM for α-amylase, and 7.70 ± 0.20 µM for α-glucosidase. Structure–activity relationship (SAR) analysis further revealed that hydroxyl substitution on the phenyl ring significantly enhances binding affinity by facilitating stronger interactions within the enzyme binding pocket. The in vitro findings were further supported by in silico investigations, including molecular docking, molecular dynamics simulations, density functional theory (DFT) analysis, pharmacophore modeling, and ADMET predictions. These complementary approaches confirmed the stability, favorable reactivity, and drug-like characteristics of the lead analogs. Overall, compounds **5**, **7**, and **10** emerged as promising candidates with superior inhibitory activity compared to acarbose, which is often associated with gastrointestinal side effects. These results suggest that the synthesized imidazo-triazole derivatives hold strong potential for further development as novel therapeutic agents for the management of type-2 diabetes mellitus.

## Figures and Tables

**Table 1:** Inhibition profile of imidazole–triazole conjugates directed at alpha-amylase and alpha-glucosidase.

S/No.	Compounds	IC_50_ = α-Amylase µM ± SEM	IC_50_ = α-Glucosidase µM ± SEM
**1**	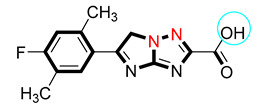	9.30 ± 0.10	9.90 ± 0.10
**2**	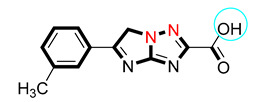	13.20 ± 0.20	14.10 ± 0.60
**3**	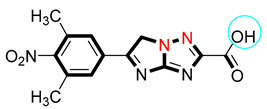	19.10 ± 0.20	19.70 ± 0.20
**4**	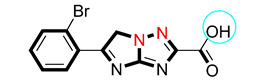	21.40 ± 0.10	22.10 ± 0.10
**5**	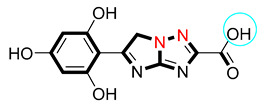	6.80 ± 0.10	7.10 ± 0.20
**6**	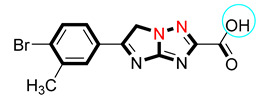	20.30 ± 0.40	21.10 ± 0.40
**7**	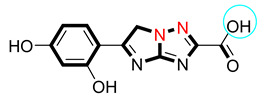	7.10 ± 0.30	7.70 ± 0.20
**8**	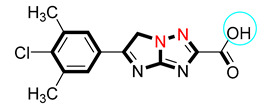	11.40 ± 0.20	12.10 ± 0.50
**9**	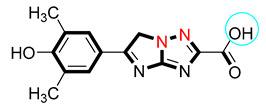	9.20 ± 0.10	9.70 ± 0.10
**10**	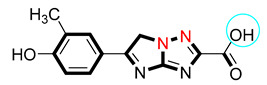	8.10 ± 0.20	8.10 ± 0.20
**11**	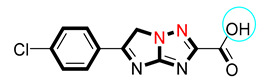	13.20 ± 0.10	13.60 ± 0.20
**12**	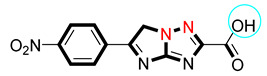	17.50 ± 0.60	18.10 ± 0.20
Standard Drug Acarbose		9.40 ± 0.20	9.80 ± 0.30

**Table 2:** Detailed docking outcomes, including type of receptor, interactions, binding distance, and docking score of potent analogs against targeted enzymes.

Compound	Receptor	Interactions	Binding Distance (Å)	Docking Score (kcal/mol)
Compound **5** in alpha-amylase complex	ARG-A-195	H-Bond	6.19	−12.44
	ARG-A-195	H-Bond	6.81	
	TYR-A-62	C–H Bond	3.53	
	TRP-A-59	Pi–Pi Stacked	4.38	
	ASP-A-300	Pi-Anion	6.81	
Compound **5** in alpha-glucosidase complex	PHE-C-439	H-Bond	5.26	−11.82
	PHE-C-439	C–H Bond	5.47	
	PHE-C-442	Pi-Alkyl	6.18	
	PHE-C-442	Pi–Pi Stacked	5.84	
	VAL-C-474	Pi-Sigma	4.93	
	VAL-C-474	Pi-Alkyl	5.51	
	TYR-A-32	H-Bond	6.62	
	ASP-A-79	H-Bond	3.06	
Compound **7** in alpha-amylase complex	ASP-A-197	H-Bond	4.27	−10.30
	TYR-A-62	Pi–Pi Stacked	4.37	
	LEU-A-165	Pi-Alkyl	5.48	
	LEU-A-165	Pi-Alkyl	5.29	
	GLN-A-63	H-Bond	4.84	
Compound **7** in alpha-glucosidase complex	ASP-A-79	H-Bond	3.76	−9.55
	ASP-A-79	H-Bond	4.83	
	GLN-C-472	H-Bond	5.85	
	TYR-A-32	C–H Bond	5.73	
	TYR-A-32	C–H Bond	6.18	
	VAL-C-474	Pi-Alkyl	4.00	
	VAL-C-474	Pi-Sigma	4.06	
	PHE-C-442	Pi-Sulfur	7.09	
Compound **10** in alpha-amylase complex	TRP-A-59	Pi–Pi Stacked	5.80	−8.76
	TRP-A-59	Pi-Alkyl	4.07	
	TRP-A-58	Pi-Alkyl	6.00	
	TYR-A-62	Pi–Pi Stacked	5.02	
	ARG-A-195	H-Bond	6.62	
	ASP-A-300	Pi-Anion	5.87	
	HIS-A-305	Pi-Alkyl	5.97	
	HIS-A-305	Pi–Pi Stacked	5.23	
Compound **10** in alpha-glucosidase complex	GLN-C-472	H-Bond	5.32	−8.00
	TYR-A-32	C–H Bond	5.69	
	LYS-C-446	Unfavorable Acceptor-Acceptor	3.95	
	PHE-C-442	Pi-Alkyl	4.71	
	PHE-C-442	Pi–Pi Stacked	5.75	
	VAL-C-474	Pi-Alkyl	5.06	
	VAL-C-474	Pi-Alkyl	4.07	
	VAL-C-474	Pi-Sigma	4.47	
	THR-C-470	C–H Bond	4.11	
	GLN-A-81	H-Bond	3.37	
	GLN-A-81	H-Bond	3.63	

**Table 3:** Detailed ADMET properties for analog **5**.

Property	Model Name	Predicted Value	Unit
Absorption	Water solubility	−2.762	Numeric (log mol/L)
	TPSA	141.06	Numeric (Å^2^)
	Caco2 permeability	−0.723	Numeric (log Papp in 10^−6^ cm/s)
	Intestinal absorption (human)	52.614	Numeric (% Absorbed)
	Skin Permeability	−2.735	Numeric (log Kp)
	P-glycoprotein substrate	Yes	Categorical (Yes/No)
	P-glycoprotein I inhibitor	No	Categorical (Yes/No)
	P-glycoprotein II inhibitor	No	Categorical (Yes/No)
Distribution	VDss (human)	−0.149	Numeric (log L/kg)
	Fraction unbound (human)	0.378	Numeric (Fu)
	BBB permeability	−1.576	Numeric (log BB)
	CNS permeability	−4.303	Numeric (log PS)
Metabolism	CYP2D6 substrate	No	Categorical (Yes/No)
	CYP3A4 substrate	No	Categorical (Yes/No)
	CYP1A2 inhibitor	No	Categorical (Yes/No)
	CYP2C19 inhibitor	No	Categorical (Yes/No)
	CYP2C9 inhibitor	No	Categorical (Yes/No)
	CYP2D6 inhibitor	No	Categorical (Yes/No)
	CYP3A4 inhibitor	No	Categorical (Yes/No)
Excretion	Total Clearance	0.499	Numeric (log ml/min/kg)
	Renal OCT2 substrate	No	Categorical (Yes/No)
Toxicity	AMES toxicity	No	Categorical (Yes/No)
	Max. tolerated dose (human)	0.541	Numeric (log mg/kg/day)
	hERG I inhibitor	No	Categorical (Yes/No)
	hERG II inhibitor	No	Categorical (Yes/No)
	Oral Rat Acute Toxicity (LD50)	2.457	Numeric (mol/kg)
	Oral Rat Chronic Toxicity (LOAEL)	2.828	Numeric (log mg/kg_bw/day)
	Hepatotoxicity	No	Categorical (Yes/No)
	Skin Sensitization	No	Categorical (Yes/No)
	T. Pyriformis toxicity	0.285	Numeric (log ug/L)
	Minnow toxicity	2.363	Numeric (log mM)

## Data Availability

Data is contained within the article or supplementary material.

## Supplementary Materials

The following supporting information can be downloaded at: https://www.mdpi.com/article/10.3390/ph18091333/s1, Figures S1–S4: Molecular interaction profile of compounds **7** and **10** with alpha-amylase and alpha-glucosidase; Figure S5: Molecular electrostatic potential and FMO analysis of compound **7**; Chart S1: ADME profile of potent analog **7**; Chart S2: DME analysis of compound **10**; Figure S6: Boiled egg diagram for analog **5**; Figures S7 and S8: Compounds **7** and **10** domain in comparison to drug like upper and lower domain characteristics; Charts S3 and S4: Molecular weight distribution and dose value distribution for analog **5**; Tables S1 and S2: Detailed ADMET properties for analog **7** and **10**; Figures S9–S11: Proton, carbon and mass spectral analysis of compound **3**; Figures S12–S14: Proton, carbon and mass spectral analysis of compound **5**; Figures S15–S17: Proton, carbon and mass spectral analysis of compound **7**; Figures S18–S20: Proton, carbon and mass spectral analysis of compound **8**.
