# Supplementary material for: Fused Imidazotriazole-Based Therapeutics: A Multidisciplinary Study Against Diabetes-Linked Enzymes Alpha-Amylase and Alpha-Glucosidase Using In Vitro and In Silico Methods
Source: Pharmaceuticals (Basel). 2025 Sep 5;18(9):1333. doi: 10.3390/ph18091333 (PMC12472753; doi:10.3390/ph18091333)
Supplement: Supplementary file 1 [file pharmaceuticals-18-01333-s001.zip › pharmaceuticals-3842086-supplementary.pdf]

Fused Imidazotriazole-Based Therapeutics: A Multidisciplinary Study Against Diabetes-Linked Enzymes Alpha-Amylase and Alpha-Glucosidase Using In Vitro and In Silico Methods

Manal M. Khowdiary * and Shifa Felemban

Department of Chemistry, Faculty of Applied Science, University College-Al Leith, University of Umm Al-Qura, Makkah 21955, Saudi Arabia

*Corresponding author: *Mmkhowdiary@uqu.edu.sa* (M.M. Khowdiary)

Supplementary Information

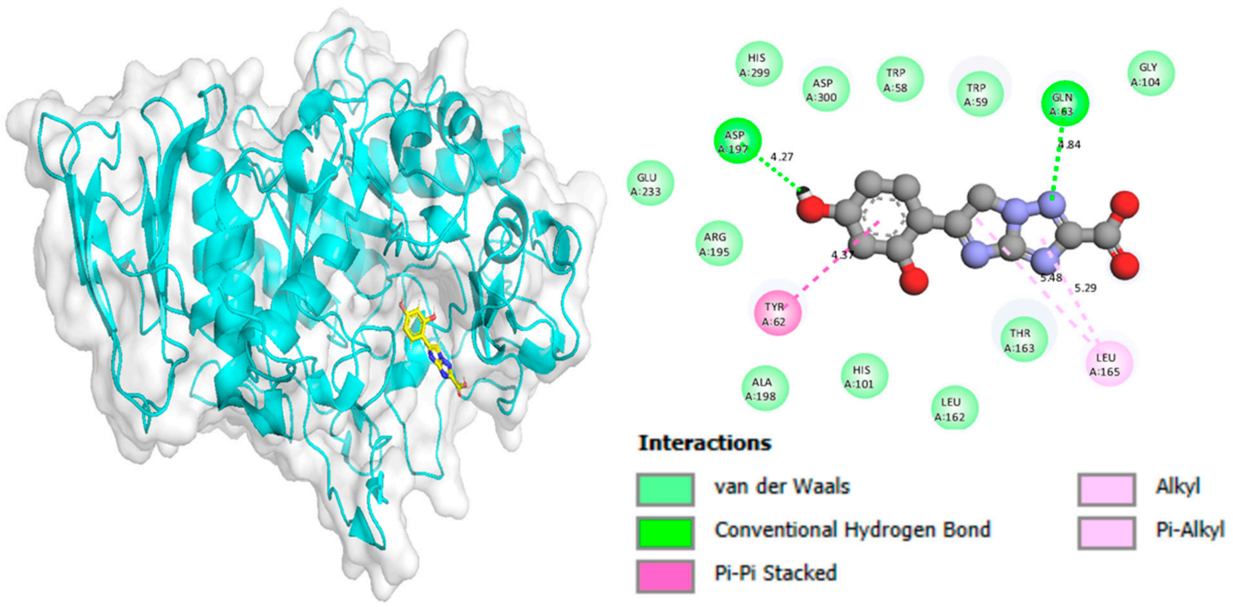

Figure-S1: Molecular interaction profile of compound **7** with alpha-amylase

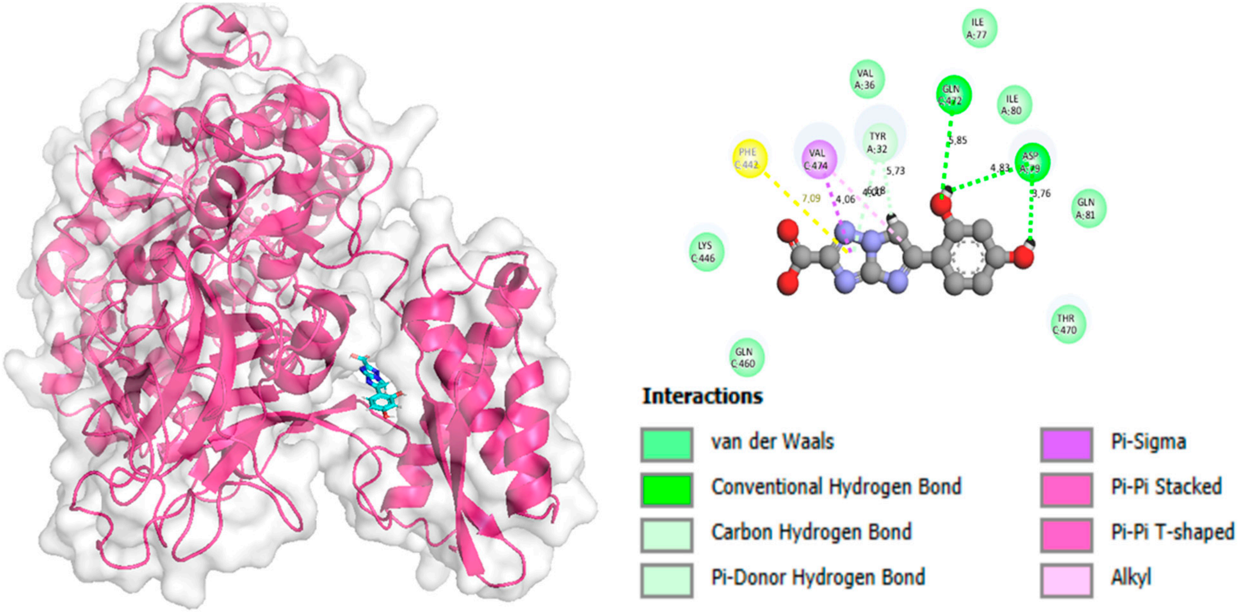

Figure-S2: Molecular interaction profile of compound **7** with alpha-glucosidase

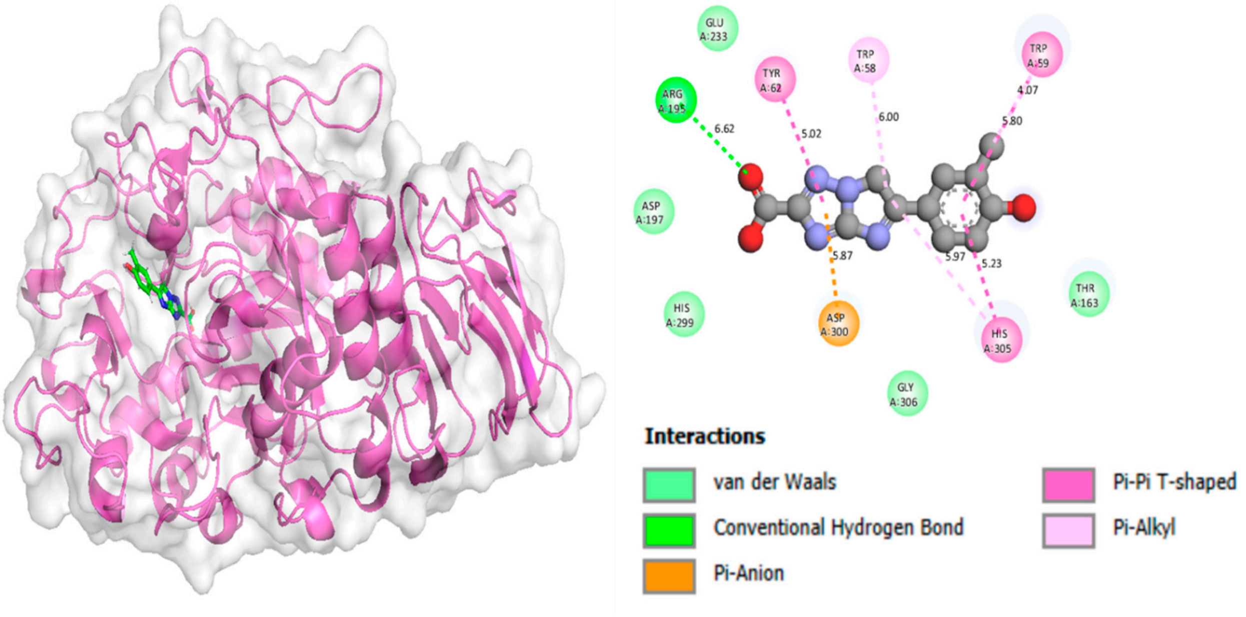

Figure-S3: Molecular interaction profile of compound **10** with alpha-amylase

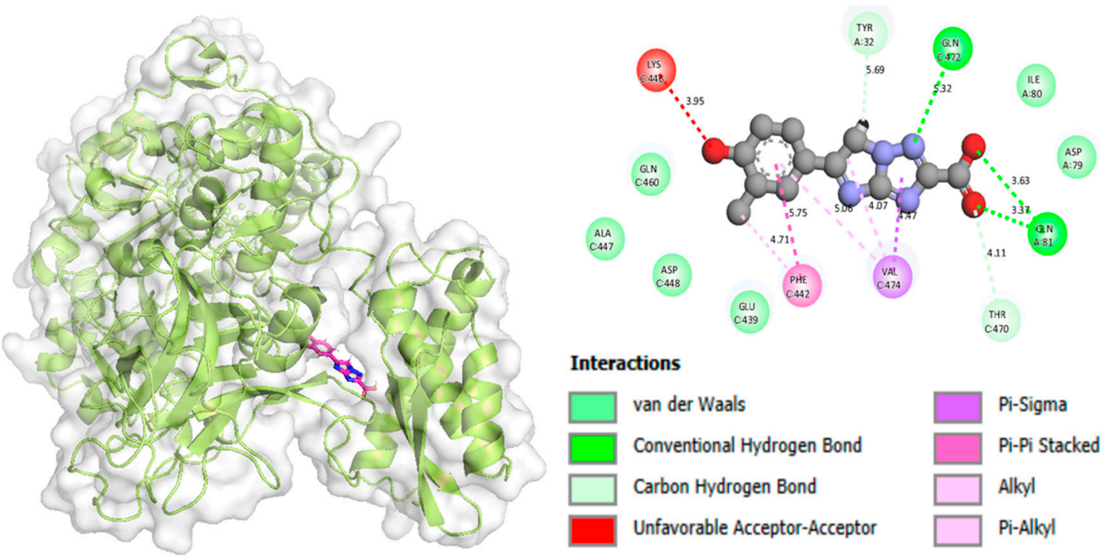

Figure-S4: Molecular interaction profile of compound **10** with alpha-glucosidase

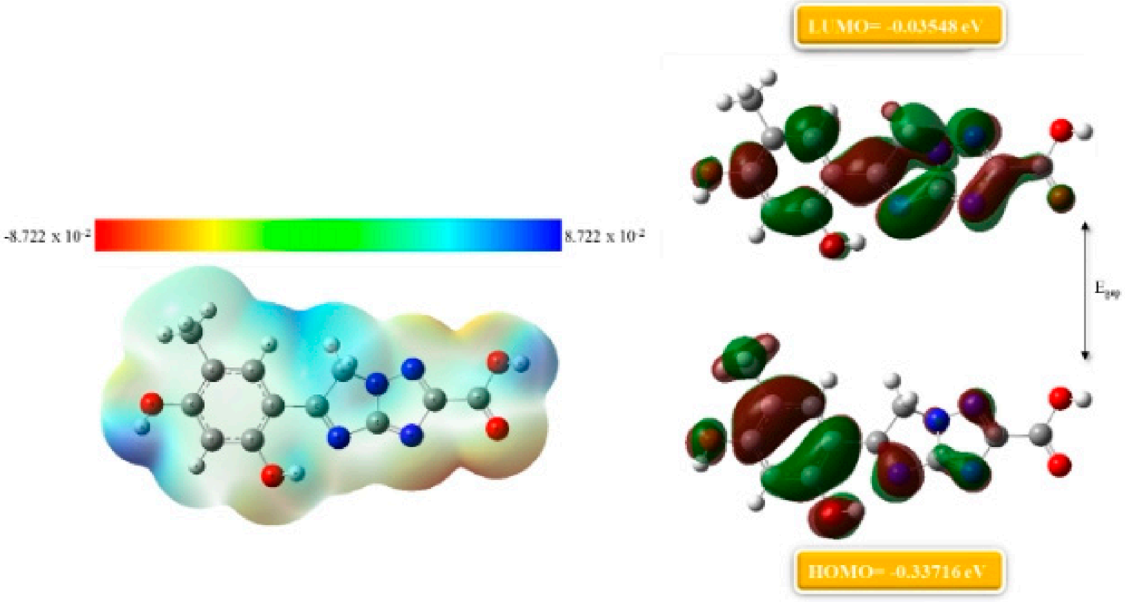

Figure-S5: Molecular electrostatic potential and FMO analysis of compound **7**

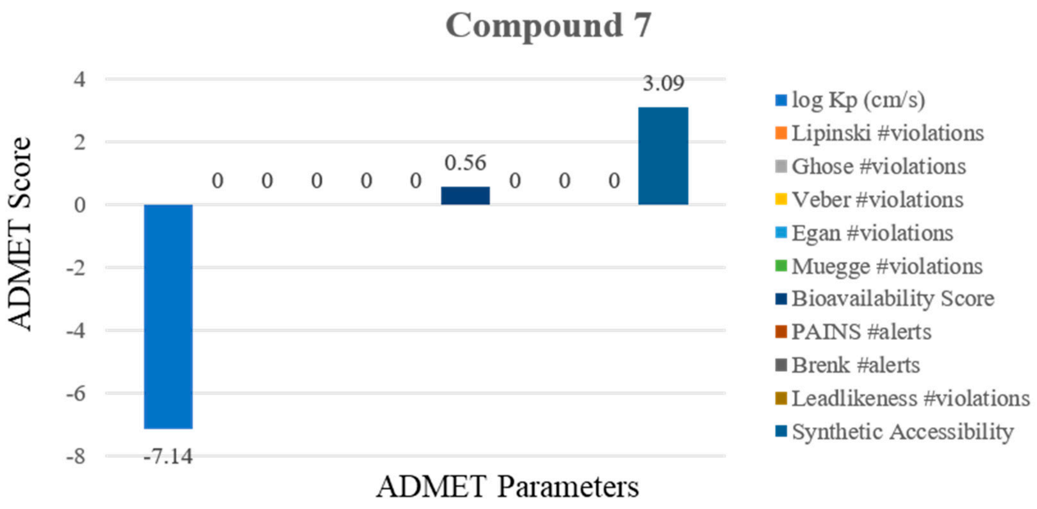

Chart-S1: ADME profile of potent analog **7**

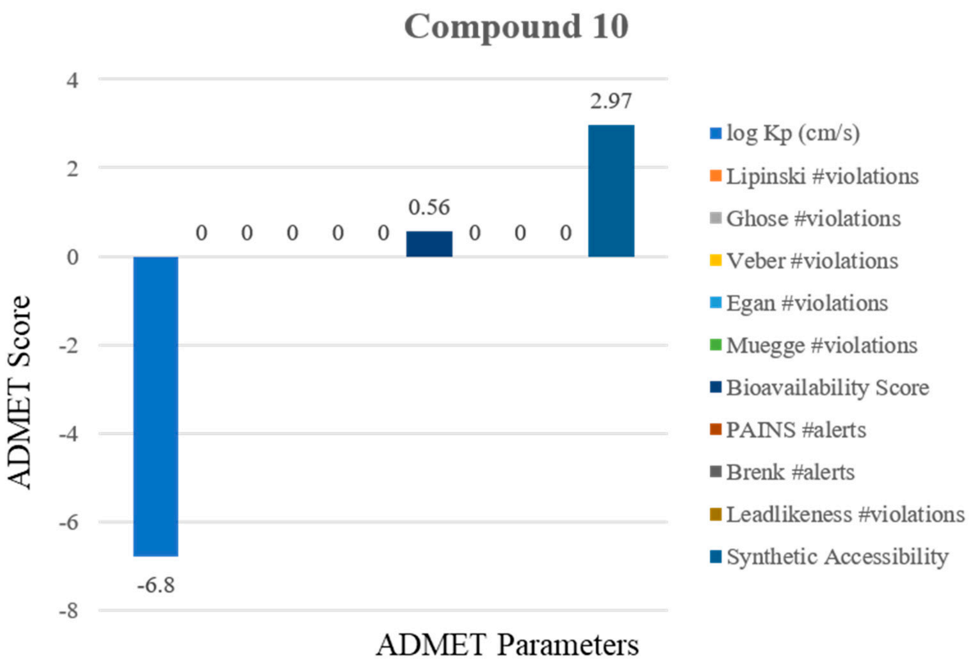

Chart-S2: ADME analysis of compound 10

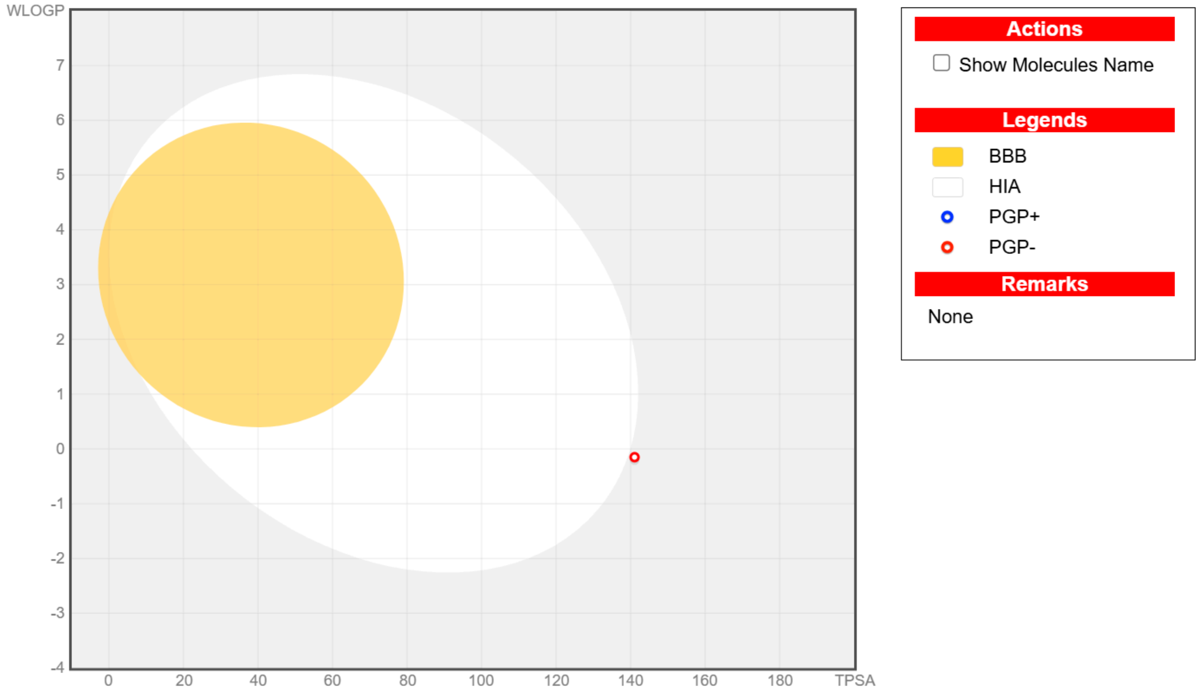

Figure-S6: Boiled egg diagram for analog **5**

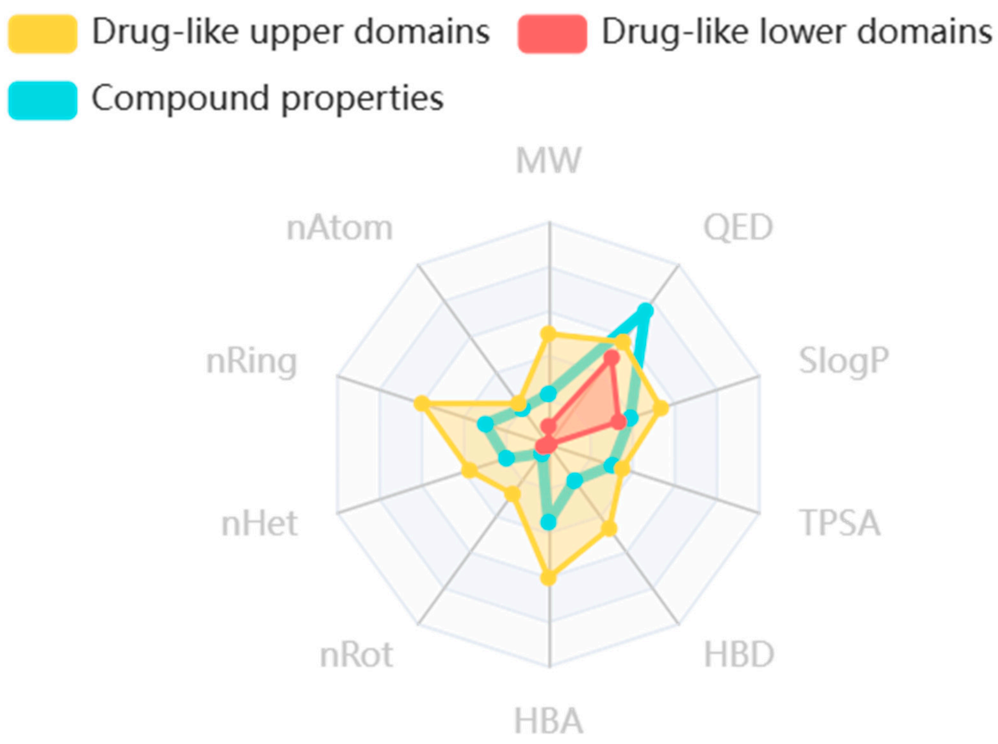

Figure-S7: Compound **7** domain in comparison to drug like upper and lower domain characteristics

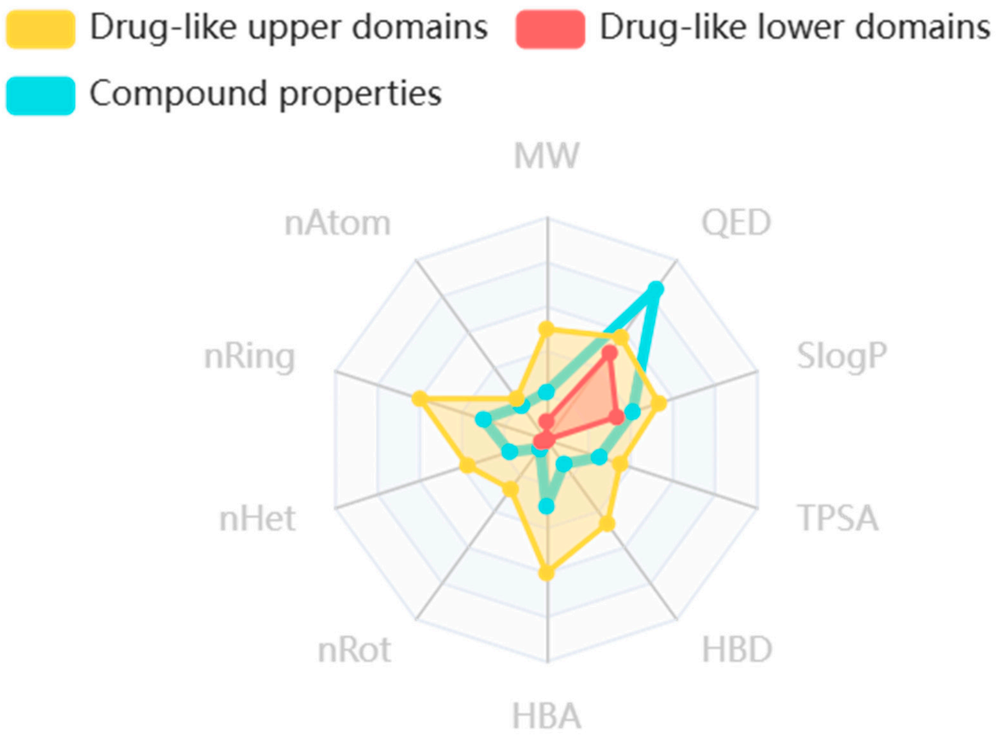

Figure-S8: Compound **10** domain in comparison to drug like upper and lower domain characteristics

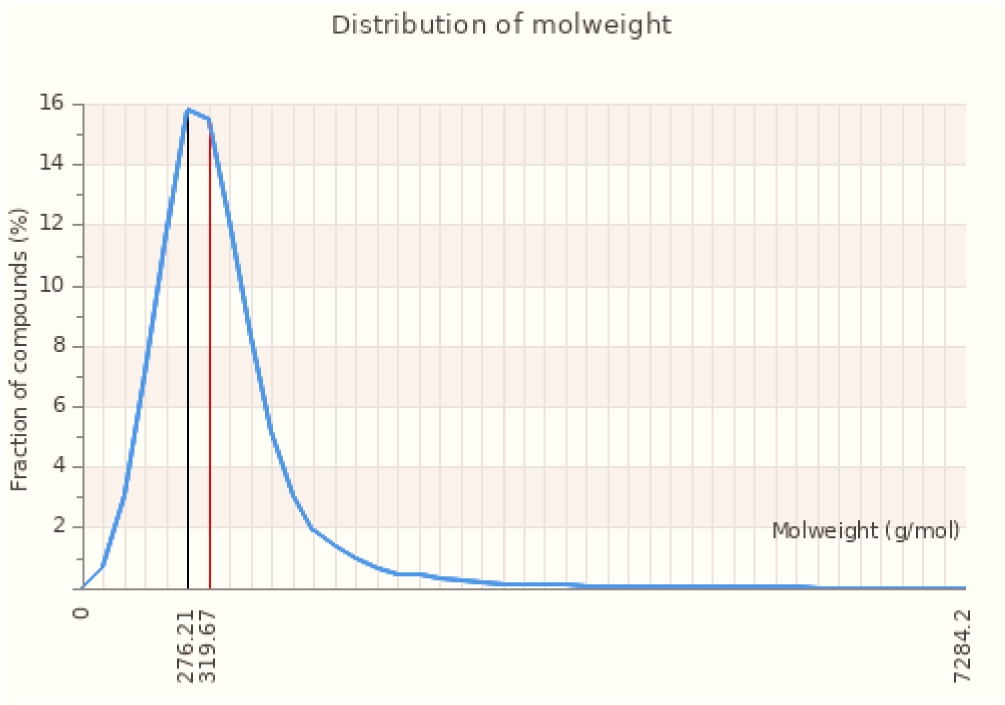

Chart-S3: Molecular weight distribution for analog **5**

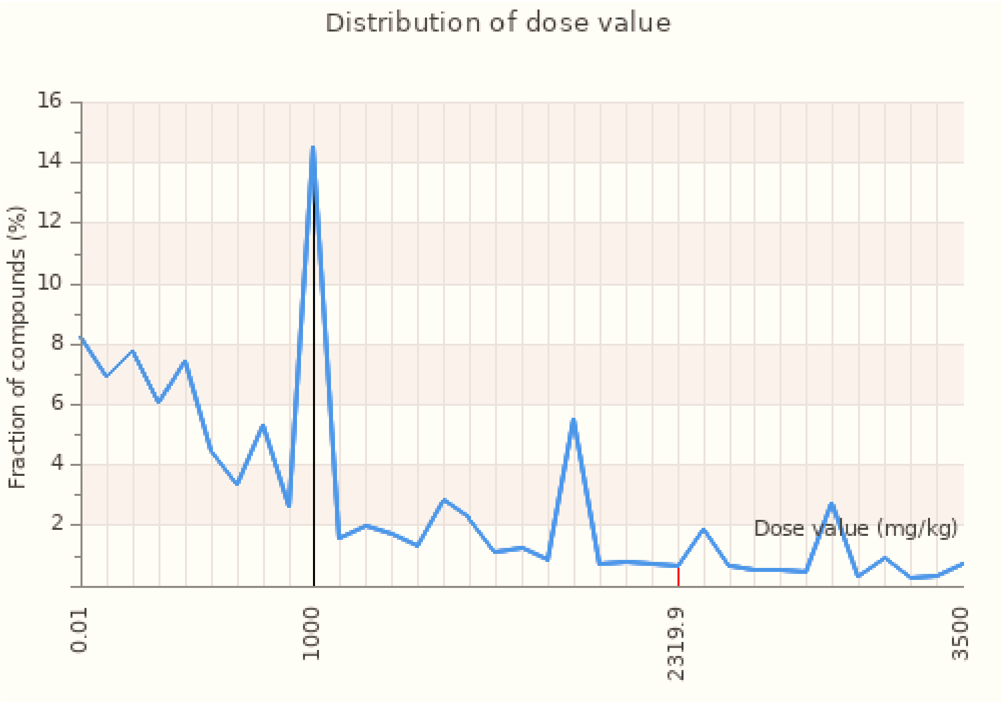

Chart-S4: Dose value distribution for analog **5**

Table-S1: Detailed ADMET properties for analog **7**

**Property**	**Model Name**	**Predicted Value**	**Unit**
Absorption	Water solubility	**-2.901**	Numeric (log mol/L)
	Caco2 permeability	**0.532**	Numeric (log Papp in 10^{-6} cm/s)
	Intestinal absorption (human)	**59.872**	Numeric (% Absorbed)
	Skin Permeability	**-2.736**	Numeric (log Kp)
	P-glycoprotein substrate	**Yes**	Categorical (Yes/No)
	P-glycoprotein I inhibitor	**No**	Categorical (Yes/No)
	P-glycoprotein II inhibitor	**No**	Categorical (Yes/No)
Distribution	VDss (human)	**-0.36**	Numeric (log L/kg)
	Fraction unbound (human)	**0.459**	Numeric (Fu)
	BBB permeability	**-1.243**	Numeric (log BB)
	CNS permeability	**-3.489**	Numeric (log PS)
Metabolism	CYP2D6 substrate	**No**	Categorical (Yes/No)
	CYP3A4 substrate	**No**	Categorical (Yes/No)
	CYP1A2 inhibitior	**No**	Categorical (Yes/No)
	CYP2C19 inhibitior	**No**	Categorical (Yes/No)
	CYP2C9 inhibitior	**No**	Categorical (Yes/No)
	CYP2D6 inhibitior	**No**	Categorical (Yes/No)
	CYP3A4 inhibitior	**No**	Categorical (Yes/No)
Excretion	Total Clearance	**0.485**	Numeric (log ml/min/kg)
	Renal OCT2 substrate	**No**	Categorical (Yes/No)
Toxicity	AMES toxicity	**No**	Categorical (Yes/No)
	Max. tolerated dose (human)	**0.745**	Numeric (log mg/kg/day)
	hERG I inhibitor	**No**	Categorical (Yes/No)
	hERG II inhibitor	**No**	Categorical (Yes/No)
	Oral Rat Acute Toxicity (LD50)	**2.228**	Numeric (mol/kg)
	Oral Rat Chronic Toxicity (LOAEL)	**2.076**	Numeric (log mg/kg_bw/day)
	Hepatotoxicity	**Yes**	Categorical (Yes/No)
	Skin Sensitisation	**No**	Categorical (Yes/No)
	T.Pyriformis toxicity	**0.247**	Numeric (log ug/L)
	Minnow toxicity	**1.884**	Numeric (log mM)

Table-S2: Detailed ADMET properties for analog **10**

**Property**	**Model Name**	**Predicted Value**	**Unit**
**Absorption**	Water solubility	**-3.419**	Numeric (log mol/L)
	Caco2 permeability	**0.635**	Numeric (log Papp in 10^{-6} cm/s)
	Intestinal absorption (human)	**64.429**	Numeric (% Absorbed)
	Skin Permeability	**-2.737**	Numeric (log Kp)
	P-glycoprotein substrate	**Yes**	Categorical (Yes/No)
	P-glycoprotein I inhibitor	**No**	Categorical (Yes/No)
	P-glycoprotein II inhibitor	**No**	Categorical (Yes/No)
**Distribution**	VDss (human)	**-0.449**	Numeric (log L/kg)
	Fraction unbound (human)	**0.515**	Numeric (Fu)
	BBB permeability	**-0.521**	Numeric (log BB)
	CNS permeability	**-3.038**	Numeric (log PS)
**Metabolism**	CYP2D6 substrate	**No**	Categorical (Yes/No)
	CYP3A4 substrate	**No**	Categorical (Yes/No)
	CYP1A2 inhibitior	**No**	Categorical (Yes/No)
	CYP2C19 inhibitior	**No**	Categorical (Yes/No)
	CYP2C9 inhibitior	**No**	Categorical (Yes/No)
	CYP2D6 inhibitior	**No**	Categorical (Yes/No)
	CYP3A4 inhibitior	**No**	Categorical (Yes/No)
**Excretion**	Total Clearance	**0.415**	Numeric (log ml/min/kg)
	Renal OCT2 substrate	**No**	Categorical (Yes/No)
**Toxicity**	AMES toxicity	**Yes**	Categorical (Yes/No)
	Max. tolerated dose (human)	**0.678**	Numeric (log mg/kg/day)
	hERG I inhibitor	**No**	Categorical (Yes/No)
	hERG II inhibitor	**No**	Categorical (Yes/No)
	Oral Rat Acute Toxicity (LD50)	**2.307**	Numeric (mol/kg)
	Oral Rat Chronic Toxicity (LOAEL)	**1.334**	Numeric (log mg/kg_bw/day)
	Hepatotoxicity	**No**	Categorical (Yes/No)
	Skin Sensitisation	**No**	Categorical (Yes/No)
	T.Pyriformis toxicity	**0.263**	Numeric (log ug/L)
	Minnow toxicity	**1.634**	Numeric (log mM)

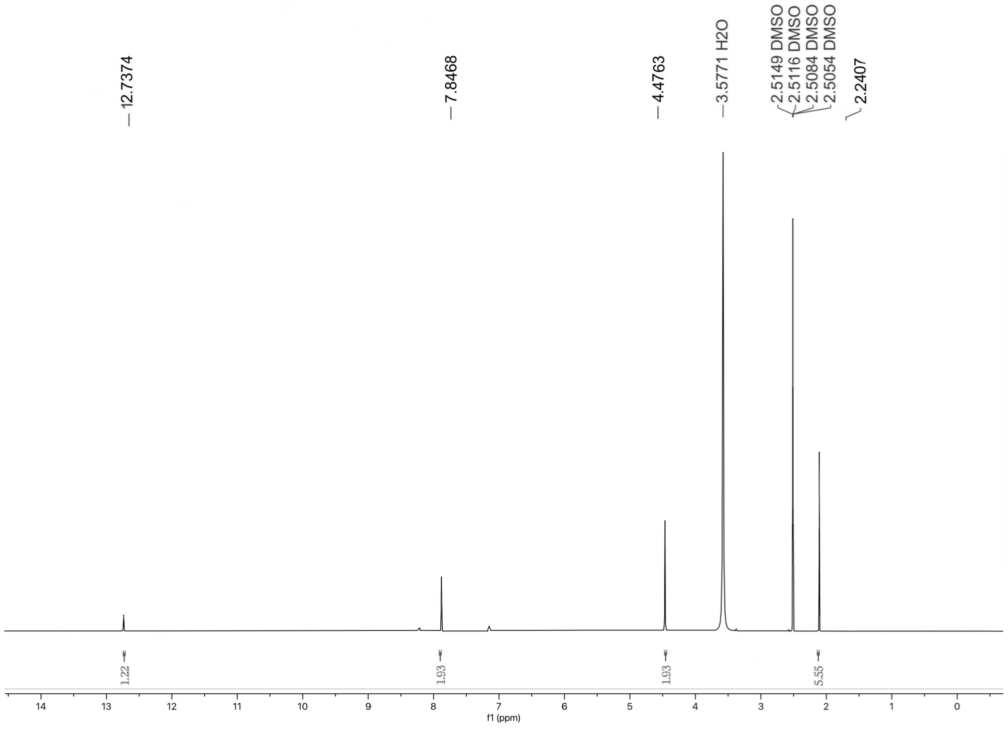

Figure-S9: Proton spectral analysis of compound **3**

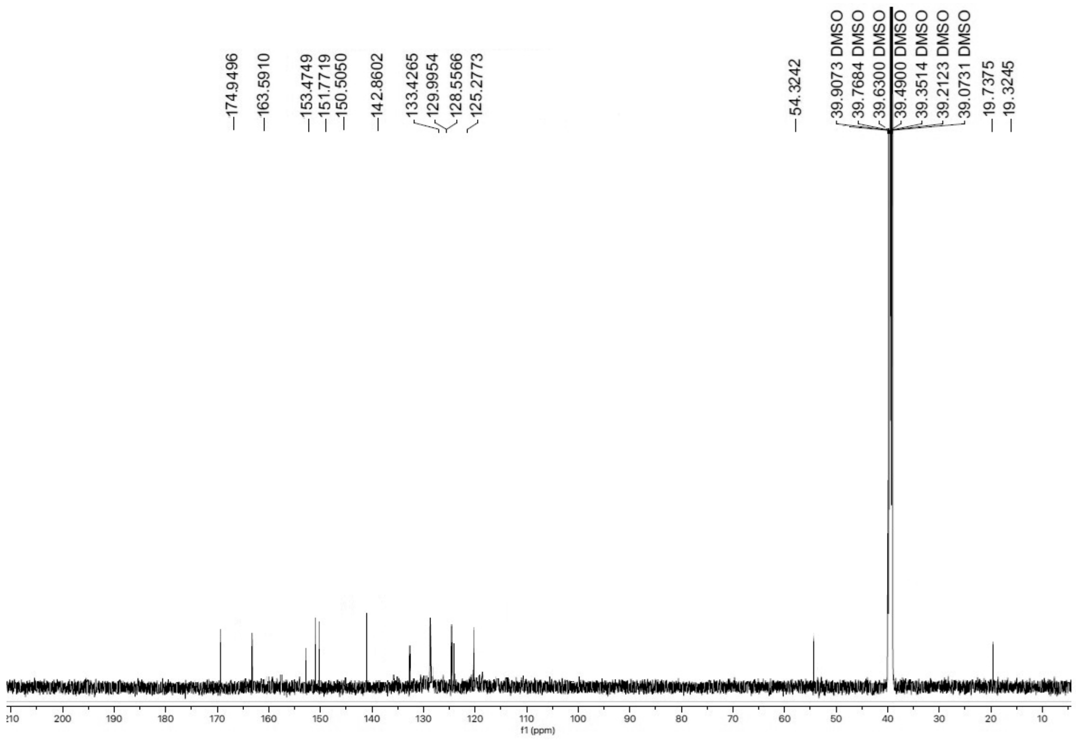

Figure-S10: Carbon spectral analysis of compound **3**

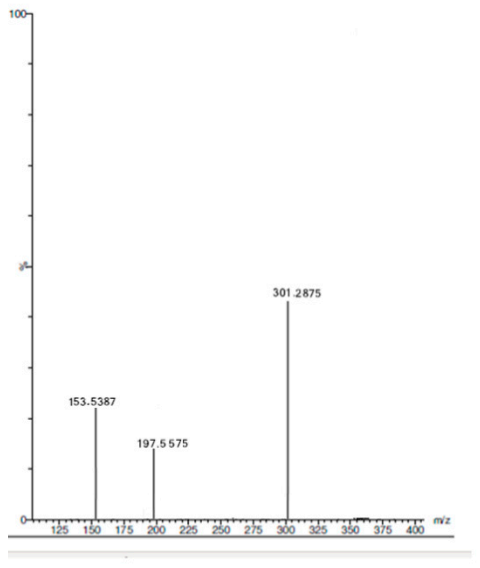

Figure-S11: Mass spectral analysis of compound **3**

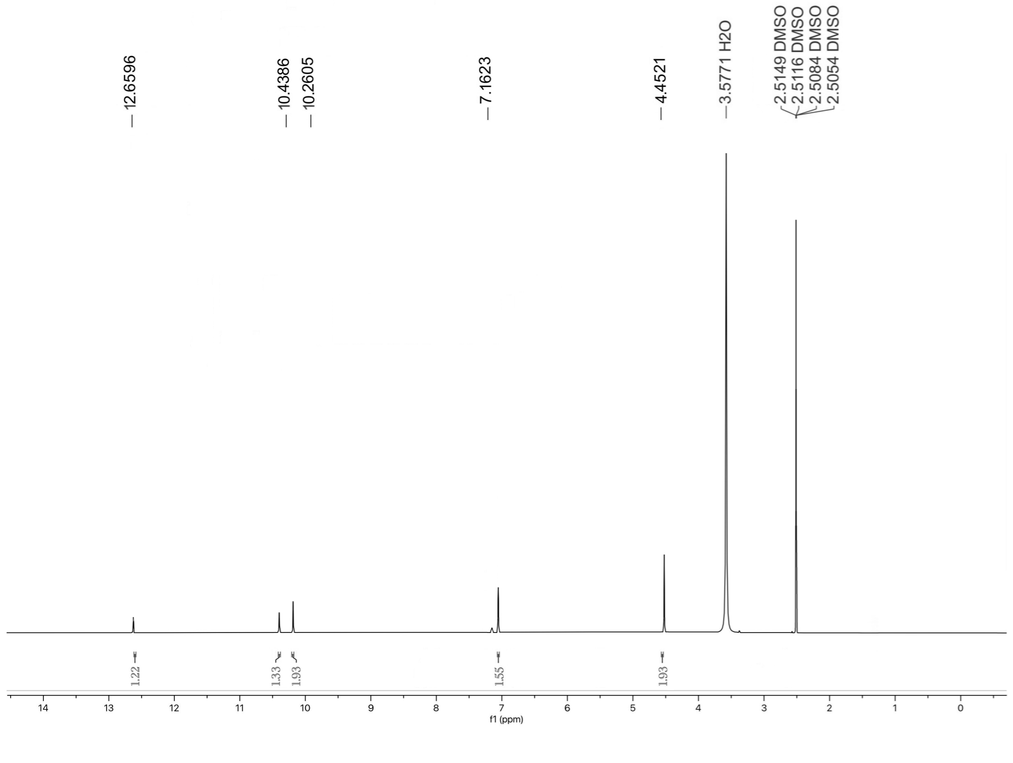

Figure-S12: Proton spectral analysis of compound **5**

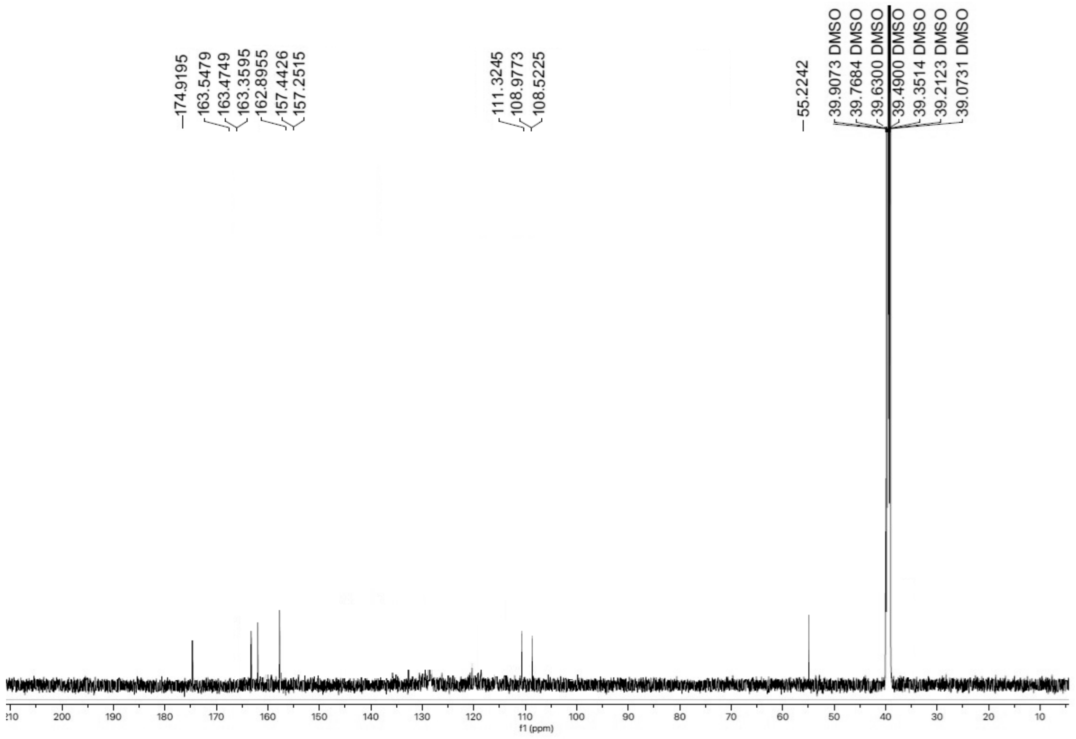

Figure-S13: Carbon spectral analysis of compound **5**

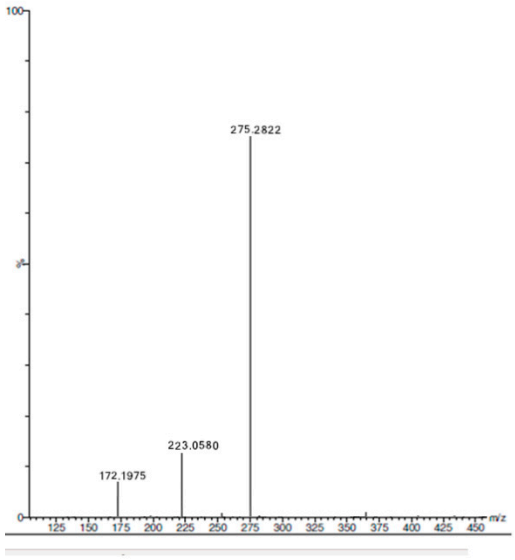

Figure-S14: Mass spectral analysis of compound **5**

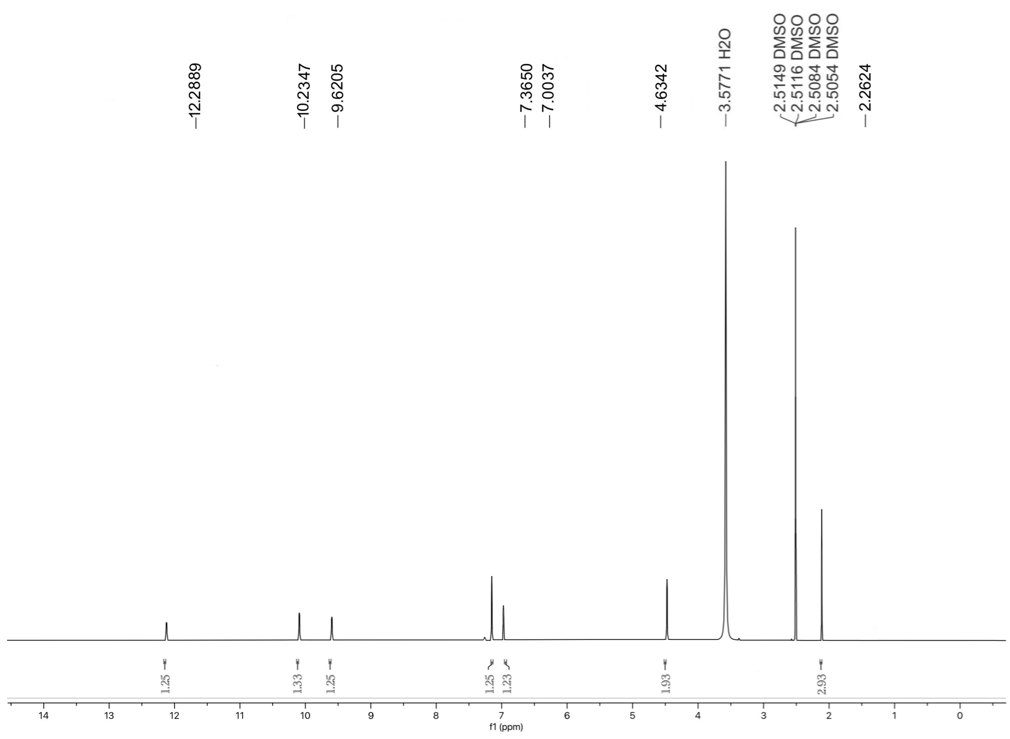

Figure-S15: Proton spectral analysis of compound **7**

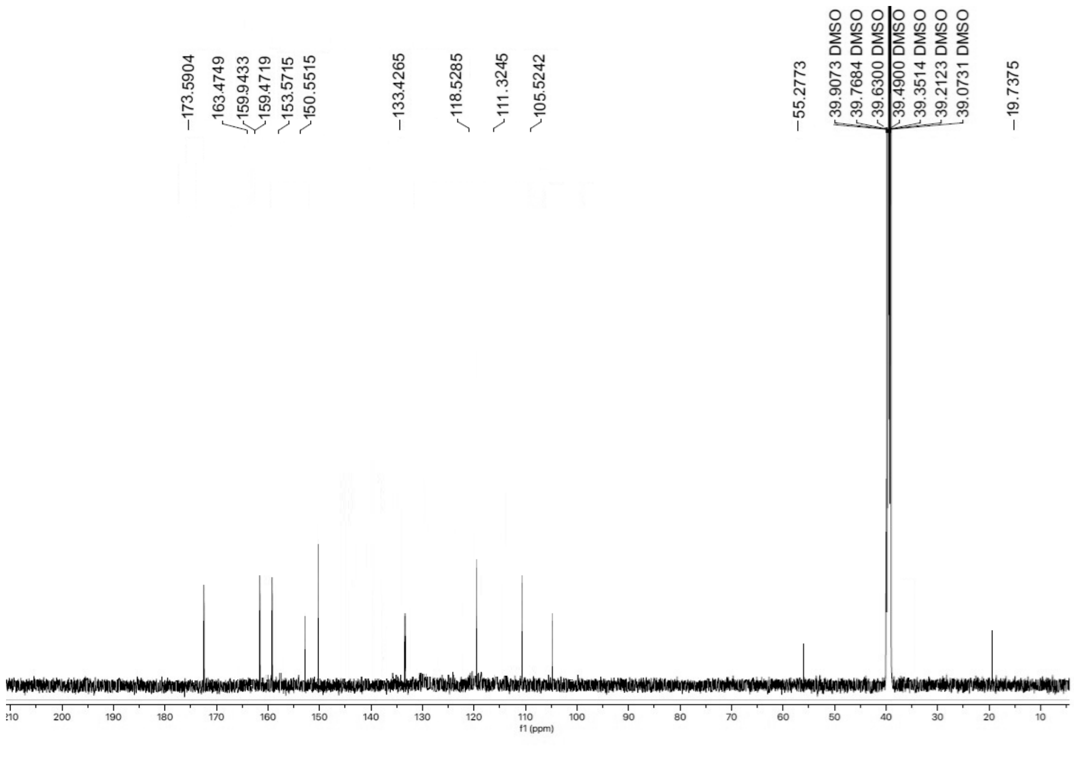

Figure-S16: Carbon spectral analysis of compound **7**

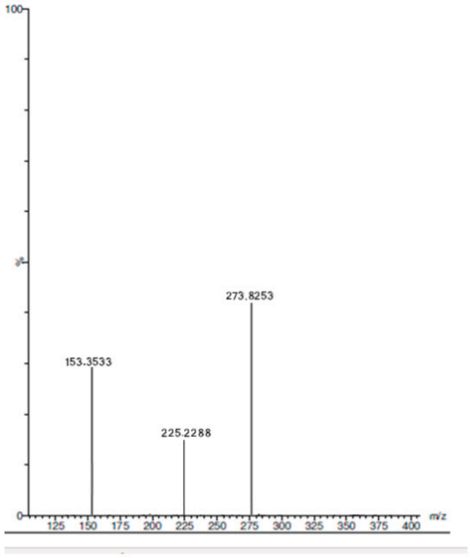

Figure-S17: Mass spectral analysis of compound **7**

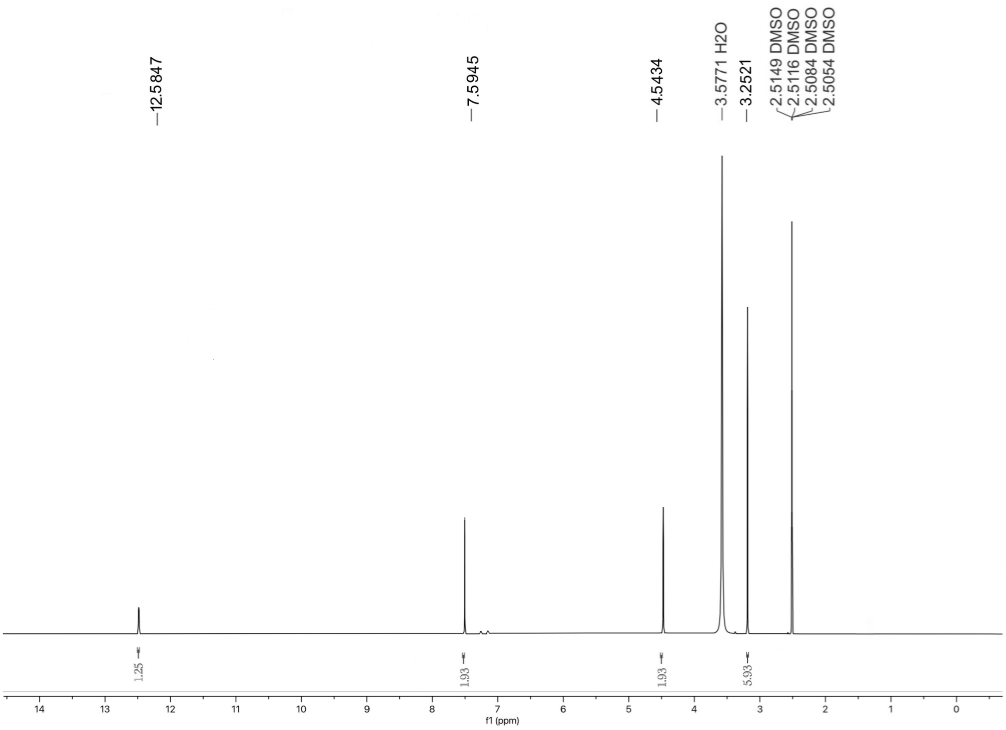

Figure-S18: Proton spectral analysis of compound **8**

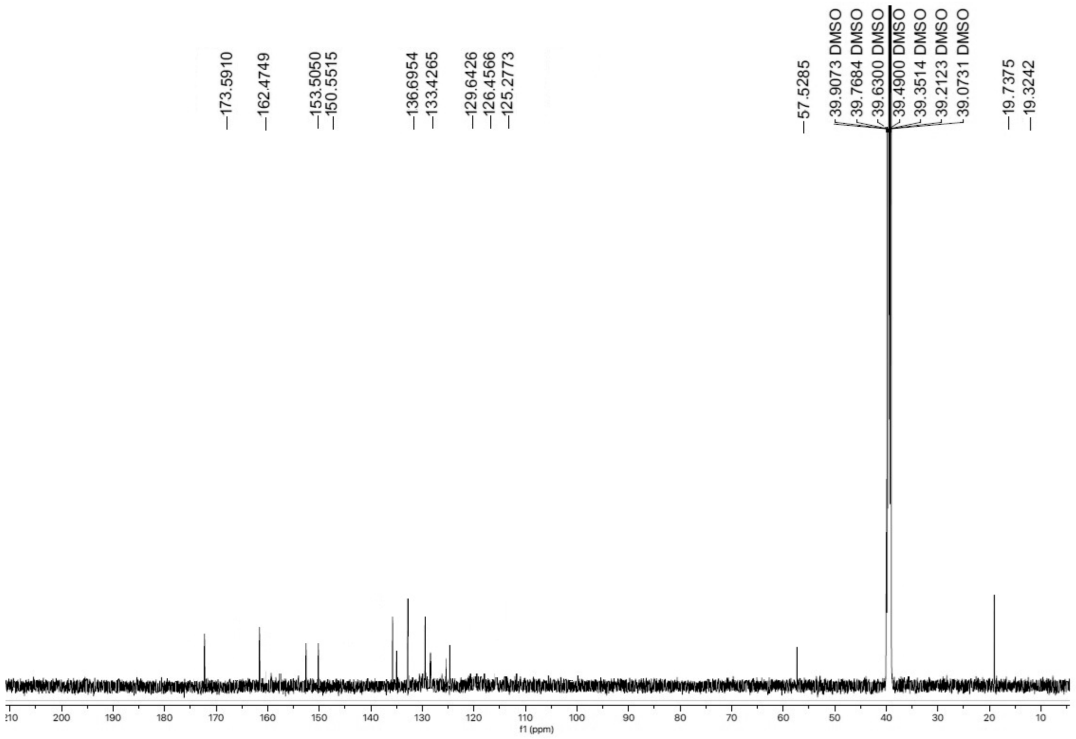

Figure-S19: Carbon spectral analysis of compound **8**

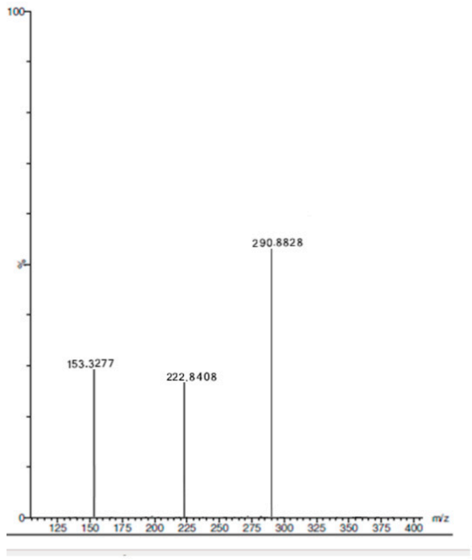

Figure-S20: Mass spectral analysis of compound **8**
